# GWAS to Identify Genetic Loci for Resistance to Yellow Rust in Wheat Pre-Breeding Lines Derived From Diverse Exotic Crosses

**DOI:** 10.3389/fpls.2019.01390

**Published:** 2019-10-30

**Authors:** Lourdes Ledesma-Ramírez, Ernesto Solís-Moya, Gabriel Iturriaga, Deepmala Sehgal, M. Humberto Reyes-Valdes, Víctor Montero-Tavera, Carolina P. Sansaloni, Juan Burgueño, Cynthia Ortiz, César L. Aguirre-Mancilla, Juan G. Ramírez-Pimentel, Prashant Vikram, Sukhwinder Singh

**Affiliations:** ^1^Departamento de estudios e investigación de Posgrado, Tecnológico Nacional de México/Instituto Tecnológico de Roque, Celaya, Mexico; ^2^Programa de mejoramiento genetico de trigo, Instituto Nacional de Investigaciones Forestales Agrícolas y Pecuarias, Campo Experimental Bajío, Celaya, Mexico; ^3^Department of Bioscience, Centro Internacional de Mejoramiento de Maíz y Trigo, Texcoco, Mexico; ^4^Departamento de Fitomejoramiento, Universidad Autónoma Agraria Antonio Narro, Saltillo, Mexico; ^5^Department of Biotechnology, Geneshifters, Pullman, WA, United States

**Keywords:** DArTseq, linked top-cross population, pre-breeding lines, yellow rust resistance, GBS, bread wheat

## Abstract

Yellow rust (YR) or stripe rust, caused by *Puccinia striformis* f. sp *tritici* Eriks (*Pst*), is a major challenge to resistance breeding in wheat. A genome wide association study (GWAS) was performed using 22,415 single nucleotide polymorphism (SNP) markers and 591 haplotypes to identify genomic regions associated with resistance to YR in a subset panel of 419 pre-breeding lines (PBLs) developed at International Center for Maize and Wheat Improvement (CIMMYT). The 419 PBLs were derived from an initial set of 984 PBLs generated by a three-way crossing scheme (exotic/elite1//elite2) among 25 best elites and 244 exotics (synthetics, landraces) from CIMMYT’s germplasm bank. For the study, 419 PBLs were characterized with 22,415 high-quality DArTseq-SNPs and phenotyped for severity of YR disease at five locations in Mexico. A population structure was evident in the panel with three distinct subpopulations, and a genome-wide linkage disequilibrium (LD) decay of 2.5 cM was obtained. Across all five locations, 14 SNPs and 7 haplotype blocks were significantly (*P* < 0.001) associated with the disease severity explaining 6.0 to 14.1% and 7.9 to 19.9% of variation, respectively. Based on average LD decay of 2.5 cM, identified 14 SNP–trait associations were delimited to seven quantitative trait loci in total. Seven SNPs were part of the two haplotype blocks on chromosome 2A identified in haplotypes-based GWAS. *In silico* analysis of the identified SNPs showed hits with interesting candidate genes, which are related to pathogenic process or known to regulate induction of genes related to pathogenesis such as those coding for glunolactone oxidase, quinate O-hydroxycinnamoyl transferase, or two-component histidine kinase. The two-component histidine kinase, for example, acts as a sensor in the perception of phytohormones ethylene and cytokinin. Ethylene plays a very important role in regulation of multiple metabolic processes of plants, including induction of defense mechanisms mediated by jasmonate. The SNPs linked to the promising genes identified in the study can be used for marker-assisted selection.

## Introduction

Wheat (*Triticum aestivum* L.) is one of the most important cereal crops with an estimated production of 736.1 million tons in 2019 (FAO, 2018). The average wheat production in Mexico is 3.3 million tons (SIAP, 2019), while the consumption is 7.17 million tons (SAGARPA, 2018). Yellow rust (YR) or stripe rust caused by the fungus *Puccinia striformis* f. sp *tritici* Eriks is one of the main phytosanitary challenges of wheat cultivation in the world, causing yield losses of 30 to 100% (Bux et al., 2012). The fungus reproduces rapidly, can travel at very large distances, has high mutation rates, and adapts to different climatic zones (Ali et al., 2017). In the presence of rust the flow of carbohydrates through the phloem to the grains is considerably reduced (Chen, 2005) due to the reduction of the photosynthetic area (Chen et al., 2015). As a result, infected plants produce fewer spikelets and form less and shriveled grains (Chen et al., 2013), which are of low industrial quality and lower food value (Afzal et al., 2008; Abdennour et al., 2018).

Seedling or race-specific resistance (*i.e.* resistance controlled by major genes) provides highly effective protection against pathogenic races (Boyd, 2005). However, the fungus can mutate into new biotypes or can produce new physiological races in a short time span to overcome seedling resistance (Singh et al., 2016). An alternative to achieve greater durability of the resistance is to develop varieties that have durable resistance based on slow rusting genes also known as adult plant resistance (APR) genes (Singh et al., 2017).

The advances in next generation sequencing technologies and high-throughput genotyping systems have revolutionized the field of plant genomics, leading to easy availability of thousands of single nucleotide polymorphisms (SNPs) in crops. Wheat has particularly benefited from these advancements, in which marker number and density have been the limiting factors for a long time (Sehgal et al., 2017). Dense sets of SNPs now available from different marker platforms [90K Illumina iselect, genotyping by sequencing (GBS), DArTseq, high‐density Affymetrix Axiom^®^ genotyping array] have significantly transformed the genetic toolkit available in wheat. It has become possible to untangle the genetic architecture of traits by genome-wide association study (GWAS), a leading approach for complex trait dissection and identification of novel and superior alleles to be utilized for breeding (Sun et al., 2017). Use of GWAS studies has allowed finding new allelic variation for a plethora of traits in crops (Suwarno et al., 2015; Pantalião et al., 2016). In wheat, GWAS has been used for various traits including resistance to a number of diseases (Crossa et al., 2007; Yu et al., 2012; Edae et al., 2014; Lopes et al., 2015, Sukumaran et al., 2015; Battenfield et al., 2016; Singh et al., 2016; Sehgal et al., 2017; Valluru et al., 2017; Singh et al., 2018). For YR, GWAS studies have led to significant advances in identification of genomic regions for both seedling resistance and APR (Rosewarne et al., 2013; Zegeye et al., 2014; Jighly et al., 2015).

The objective of this research was to identify genetic loci associated with resistance to YR in pre-breeding lines (PBLs) developed at International Maize and Wheat Improvement Center (CIMMYT). These lines were derived by a three-way crossing scheme (exotic/elite1//elite2) among CIMMYT’s 25 best elites and exotics (synthetics, landraces) from gene bank (Singh et al., 2018).

## Materials and Methods

### Plant Material

Out of an initial set of 984 PBLs (Singh et al., 2018), 419 were selected for the present study. Initially 984 PBLs were evaluated under heat (sowing delayed by 3 months) and drought (two irrigations were applied) stresses in Obregón, Sonora, Mexico. The outstanding 419 PBLs were selected for evaluation in this study for YR resistance. Of 419, 126, 69, 66, 22, and 21 have KAUZ/5/KACHU, KACHU/3/BAJ#1, AMAD*2/KIRITATI, BAJ#1/4/SUP152, and NAV/KACHU in their pedigrees, respectively. The remaining 115 PBLs have different lines in their pedigrees, which are detailed in **Supplementary Table 1**. The most frequent female parent was *Aegilops squarrosa* followed by KACHU in 419 lines.

### Phenotypic Evaluation

The 419 PBLs were evaluated in an experimental alpha lattice design with two replications in five locations in Mexico. Three of these locations were Villagrán, Guanajuato (20°33′10.20″ N, 101°4′38.84″ W, 1750 masl, 17.4°C t m, 8.4°C t min, 26.3°C t max, and 10.4 mm), Celaya, Guanajuato (20°34′44.89″ N, 100°49′9.51″ W, 1764 masl, 17.8°C t m, 8.5°C t min, 27.2°C t max, and 62.6 mm), and La Barca, Jalisco (20°17′26.38″ N, 102°32′44.96″ W, 1542 masl, 18.33°C t m, 8.25°C t min, 28.5°C t max, and 16.95 mm) where evaluations were done in fall–winter (FW) cycle of 2015–2016. The remaining two locations were Texcoco, State of Mexico (19°26′42.67″ N, 98°54′2.00″ W, 2250 masl, 14.0°C t m, 8.2°C t min, 21.4°C t max, and 369 mm) and Nanacamilpa, Tlaxcala (19°29′41.10″ N, 98°32′11.03″ W, 2727 masl, 16.62°C t m, 9.72°C t min, 23.54°C t max, and 112.56 mm) where evaluations were done in spring–summer (SS) cycle of 2016.

In the FW cycle of 2015–2016, the sowing was done in blocks of 20 experimental plots with each plot consisting of two rows separated by 0.75 m. The blocks were separated by 1 m, in which the susceptible variety ‘Morocco’ was planted in the form of hill plots and was inoculated with MEX14.191 and MEX10.44 races. To ensure sufficient and homogenous distribution of *Pst* across the experimental trial, seeds of ‘Morocco’ were also planted as border rows all around the trial. The sowing density of experimental plots was 46 kg ha^−1^. A total of five irrigations were applied at 0, 35, 65, 85, and 105 days after sowing and plots were fertilized with the dose 240-60-00 N-P-K; all the phosphorus and half of the nitrogen at sowing and the rest in the first auxiliary irrigation. For inoculation, urediniospores were collected from previous cycles with a mechanical collector and kept at –55°C in a freezer. Before use, frozen urediniospores received a thermal shock with water at 60°C for 7 min and then rehydrated for 4 h in a humid chamber (Mariscal et al., 2009). A suspension was made of hydrated urediniospores with water and an emulsifier at a concentration of 1 × 10^6^ ml^–1^ to be injected in the stems of the susceptible cultivar Morocco.

In the SS cycle of 2016, lines were sown in rows of 40 hill plots using 9 g per plot density. Seeds of variety ‘Morocco’ were sown only as border rows all around the trial. Plots were fertilized with the formula 120-60-00 N-P-K at sowing and no irrigation was applied. Crops were allowed to develop under natural rainfall conditions and under natural epidemic conditions in Texcoco and Tlaxcala, where races MEX10.44, MEX14.191, MEX14.146, and MEX14.141 predominate (Huerta-Espino et al., 2015). The most frequent *Pst* race in these locations is MEX14.191 (Huerta-Espino et al., 2015). The avirulence/virulence formula of these races is presented in **Supplementary Table 2**.

Rust scores were taken according to the modified Cobb scale (Peterson et al., 1948). The percentage of final severity of the lines was considered when the Morocco variety reached to the stage of 100% leaf damage. On the basis of 0–100 scale, accessions were grouped as highly resistant to resistant (0–20), intermediate (21–50), and susceptible to highly susceptible (51–100).

### Analysis of Phenotypic Data

Best linear unbiased predictors (BLUPs) were calculated from two replications data for each of the five environments separately and across combined environments using Meta-R program V.6.0 (Alvarado et al., 2016). The following the mixed linear model was used: “*y = Xr + Za + Wb + e*”, where *y* is the response variable, *X*, *Z*, and *W* are incidence matrices for replicate, genotype, and block nested in replicate effects, respectively; *r*, *a*, and *b* are the effects of replicate, lines, and block nested in replicate, respectively. Line and block effects were considered as random effects. *e* is the experimental error, which follows a normal distribution with zero mean and variance. The variance components were used for calculating broad sense heritability (*h*^2^) for traits in each location and across combined locations. For individual location, *h*^2^ was calculated as follows:

$$h^{2}{}_{\mathrm{loc}}=\;V_{g}/\text{ (}V_{g}+\;V_{\text{err}}/r\text{)}$$

where *V*_g_ is genotypic variance and *V*_err_ is the error variance and *r* = number of replications in a location. For combined locations, *h*^2^ was calculated as follows:

$$h^{2}{}_{\mathrm{comb}}=\;V_{g}/\text{ ((}V_{g}+\text{ (}V_{g\;x\;e}/n\text{)}+(\;V_{\text{err}}/\mathrm{nr})\text{)}$$

where *V*_g_ is genotypic variance, *V*_g×e_ is genotype × environment interaction variance, *V*_err_ is the error variance, *n* = number of environments, and *r* = number of replications.

Histograms showing the dispersion of disease scores of the 419 lines were made for each location with a script executed in R version 3.5.1.

### Genotyping

Genomic DNA was extracted from fresh leaves collected from the 419 individuals using the CTAB (cetyltrimethylammonium bromide) method but modified as described in Dreisigacker et al. (2016) in CIMMYT laboratory manual. DNA quality and concentration were determined by electrophoresis in 1% agarose gel. The samples were genotyped by DArTseq technology (Sansaloni et al., 2011) in the Genetic Analysis Service for Agriculture (SAGA) with current headquarters at CIMMYT, El Batan, Texcoco, Estado de Mexico, using 30 μl of DNA (80–100 ng μl^−1^) in 96-well plates. The FASTQ files (full reads of 77 bp) were quality filtered using a Phred quality score of 30, which represent a 90% of base call accuracy for at least 50% of the bases. More stringent filtering was also performed on barcode sequences using a Phred quality score of 10, which represent 99.9% of base call accuracy for at least 75% of the bases. A proprietary analytical pipeline developed by DArT P/L was used to generate allele calls for SNP and presence/absence variation (PAV) markers. Then, a set of filtering parameter was applied to select high quality markers for this specific study. One of the most important parameters is the average reproducibility of markers in technical replicates for a subset of samples which was set at 99.5%. Another critical quality parameter is call rate. This is the percentage of targets that could be scored as “0” or “1”, the threshold was set at 50%. SNPs with missing data more than 20% and minor allele frequency <0.05 were eliminated. To obtain physical positions of SNPs, sequence reads of the SNPs were blasted to the reference genome of RefSeq V.1.0 with E value <10^−8^ and identity >95%.

### Population Structure and Linkage Disequilibrium (LD) Analysis

To determine the population structure fastStructure v.1.0 (Raj et al., 2014) was used using the Bayesian clustering method. The parameters set were 10,000 burning cycles and 10,000 iterations of Monte Carlo Markov Chains using the admixture model. Cluster values (*K*) ranging from 2 to 10 were chosen and five independent runs were conducted for each *K*. A zip archive containing all of the results-f files was created and used as an input in Structure Harvester program to estimate delta *K* (∆*K*). Additionally, the principal component analysis (PCA) was performed with the TASSEL software (Trait Analysis by Association, Evolution and Linkage version 5.2.50), using 22,415 high quality and filtered DArTseq markers (missing data <30% and minor allele frequency >0.05). The population structure was corroborated by a cladogram made with the TASSEL program. The kinship matrix was also estimated using the 22,415 markers using TASSEL. The PCA was drawn using the rgl package in R. GAPIT version 2.0 was used to obtain correlation estimates of the frequency of the squared allele of LD (*r*^2^) for all pairwise comparisons. Pattern of LD decay was visualized by plotting pair-wise *r*^2^ values against the distance (cM) between markers for A, B, and D genomes separately and for whole genome. A smooth line was fit to the data using second-degree locally weighted scatterplot smoothing (LOESS; Breseghello and Sorrells, 2006) as implemented in SAS. For the LOESS estimation of LD decay, genetic distance was estimated as the point where the LOESS curve first crosses the baseline *r*^2^ of 0.1.

### Haplotype Block Construction

Haplotype blocks were generated considering the *D*′ linkage unbalance parameter using the R script described in Gabriel et al. (2002). When many markers had the same genetic position, only the first marker of those groups was taken. We calculated *D*′ 95% confidence intervals between SNPs and each comparison was categorized as “strong LD”, “inconclusive”, or “strong recombination”. A haplotype block was created if 95% of the comparisons in one block were in “strong LD”. For two or more SNPs to be classified in “strong LD”, the minimum lower and upper confidence interval values were set to 0.7 and 0.98, respectively. The haplotypes were displayed as blocks of marker numbers and alleles. They were named as combinations of the prefix “H” followed by a number representing the chromosome, a point, and then a number that represents a particular allelic combination.

### Association Analysis for Resistance to YR

The phenotypic BLUPs of each line were used for the association analysis using TASSEL program (version 5.2.50). A mixed linear model was used with PCA as a fixed variate and kinship as random. The quality of the association was analyzed by drawing Q–Q plots (**Supplementary Figure 1**). To declare significant associations, the criterion of false discovery rate was used and it was calculated using package Q-VALUE in R. The associations were represented by drawing Manhattan graphs in TASSEL.

### *In Silico* Analysis

*In silico* analysis of the significant loci was conducted in Ensembl Plants sequence database (https://plants.ensembl.org/Triticum_aestivum/Info/Index). Based on BLAST in the server, quantitative trait loci (QTL) were located on short and long arms of chromosomes and candidate gene hits were reported.

## Results

### Phenotypic Evaluation

**Figure 1:**
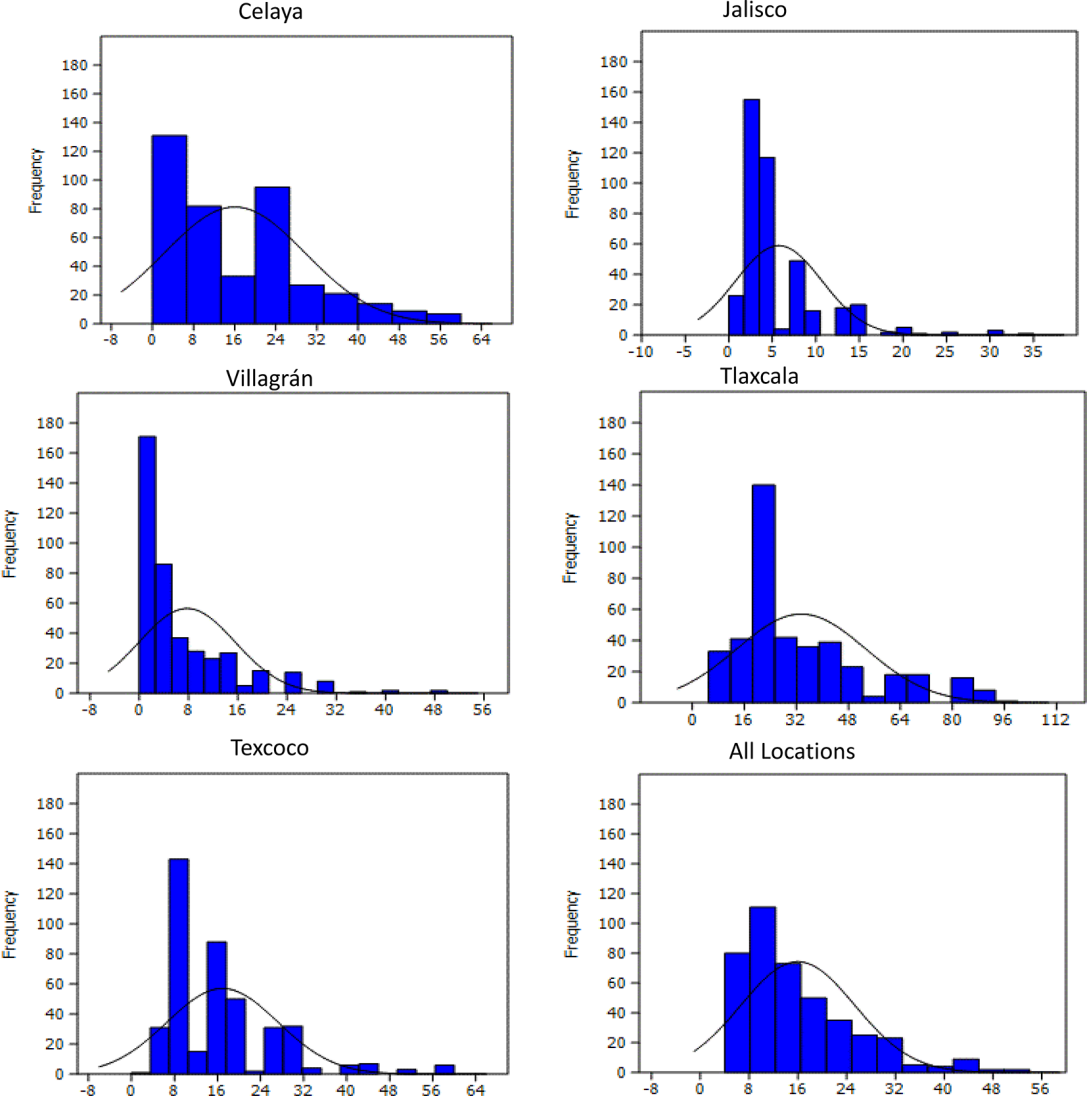
Frequency distribution of yellow rust severity (%) in 419 PBLs evaluated during the FW 2015–2016 and SS 2016 cycles in five locations of Mexico.

The minimum and maximum YR scores for all individual locations and across five locations are listed in **Supplementary Table 3**. Wide variation occurred among PBLs in all environments (**Figure 1**), with scores ranging from 0 to 70, 0 to 35, 2.5 to 70, 5 to 100, and 0 to 50% in Celaya, Jalisco, Texcoco, Tlaxcala, and Villagrán, respectively. A normal distribution of the trait was observed at all locations. 72.7, 92.3, 78.2, 33.1, and 93.5% of the lines were resistant, 15.5, 1.6, 10.2, 51.3, and 0.4% displayed intermediate reaction, and 11.6, 6.1, 11.4, 15.5, and 6.1% were moderately susceptible to very susceptible (51 to 100%). ANOVA analysis showed significant variation in lines at all locations (**Supplementary Table 3**). The zero estimates of genotype × environment interactions indicate stability of genotypic performance across environments. Location-wise heritability was high; ranging from 0.93 in Texcoco to 0.98 in Jalisco (**Supplementary Table 3**) with 0.84 across combined locations.

### Genotyping

51,232 SNPs and PAV were generated across 419 lines after allele calling, of which 22,415 filtered SNPs were utilized for further analysis. PAV markers were not used in any analysis. 6,838 SNPs were found in genome A, 7,605 in genome B, and 7,972 in genome D. The chromosome with the highest number of SNPs was 7D with 1,718 SNPs, followed by chromosome 2B with 1,453 SNPs. Chromosomes with fewer SNPs were 6A with 790, 4D with 635, and 4B with 603 SNPs. A total of 591 genome-wide haplotype blocks were obtained, of which 253 were detected in genome A, 260 in genome B, and 78 in genome D. The number of SNPs in haplotype blocks ranged from 2 to 8.

### Population Structure

**Figure 2:**
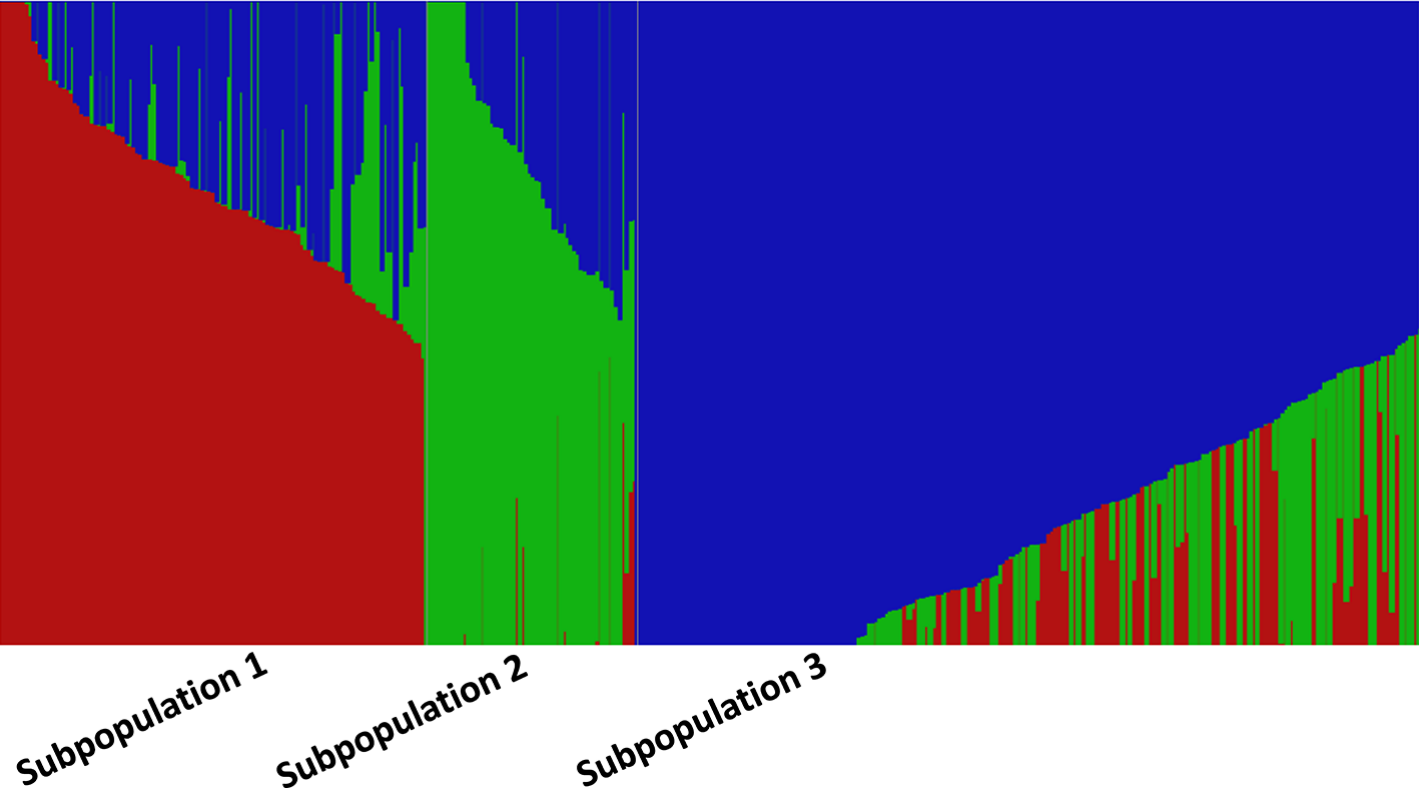
fastStructure analysis showing three subpopulations at *K* = 3. Each accession is represented by a thin vertical line, which is partitioned into three colored segments representing estimated membership probabilities (*Q*) of the individual to the three clusters.

**Figure 3:**
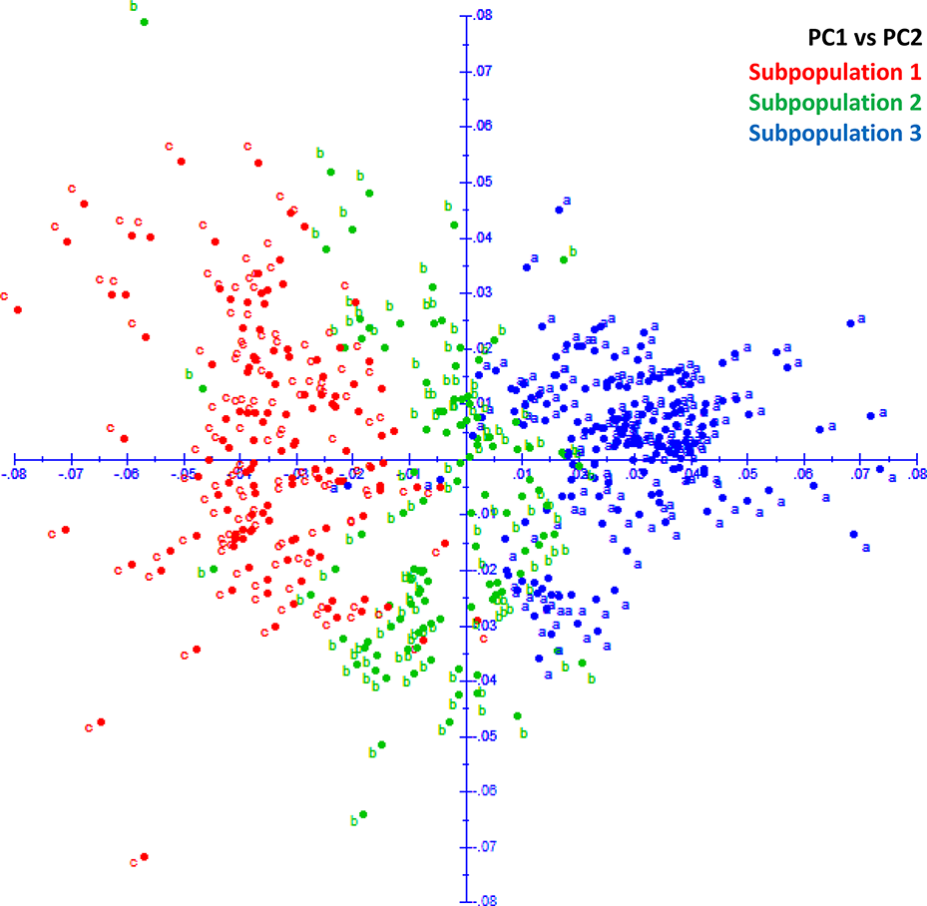
PCA plot showing three groups corresponding to three subpopulations in STRUCTURE analysis.

The fastStructure analysis showed three subpopulations in the panel determined by the best *K* (*K* = 3) by method described in Evanno et al. (2005). Subpopulation 1 (SP 1) with 131 lines shared pedigrees Baj#1, Reedling#1, Villa Juarez F2009, and Tacupeto F2001, subpopulation 2 (SP 2) with 110 lines shared pedigrees Seri.1b//Kauz/Hevo/3/Amad*2/4/Kiritati; and subpopulation 3 (SP 3) with 178 lines sharing pedigrees Fret2*2/4/Sni/Trap#1/3/Kauz*2/Trap//Kauz/5/Kachu. PCA also showed three groups, which corresponded to the three sub-populations revealed by fastStructure analysis (**Figure 2**). A PCA drawn from three PCs shows three groups akin to three sub-populations (**Figure 3**). A neighbor joining cladogram also confirmed the three groups obtained in PCA and fastStructure analysis (**Supplementary Figure 2**).

### LD Decay

**Figure 4:**
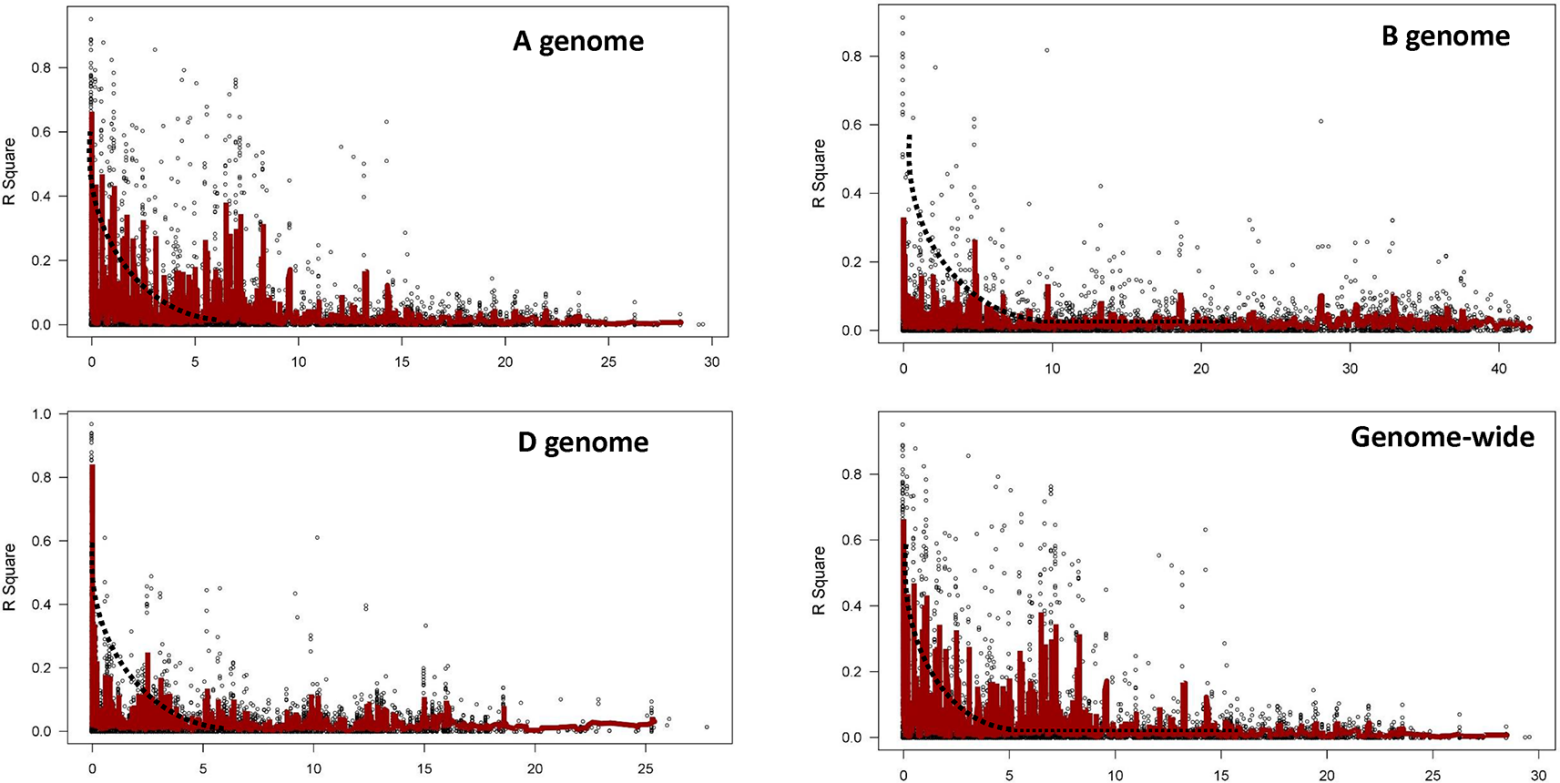
Linkage disequilibrium (LD) decay plot for the three genomes and for whole genome based on 22,415 markers.

LD was estimated by calculating the squared allele frequency correlation (*r*^2^) among all possible pairs of markers for each of the 21 chromosomes. Obtained *r*^2^ values were then plotted against genetic distance (cM) for each of the three genomes separately and across whole genome (**Figure 4**). LD decayed at 2.5, 5.0, and 2.5 cM for A, B, and D genomes, respectively at cut off *r*^2^ = 0.1, while for whole genome, decay was observed at 2.5 cM. Based on this average LD decay, size of QTL was estimated *i.e.* all significant SNPs within 2.5 cM were considered as part of the same QTL.

### Association Mapping

**Figure 5:**
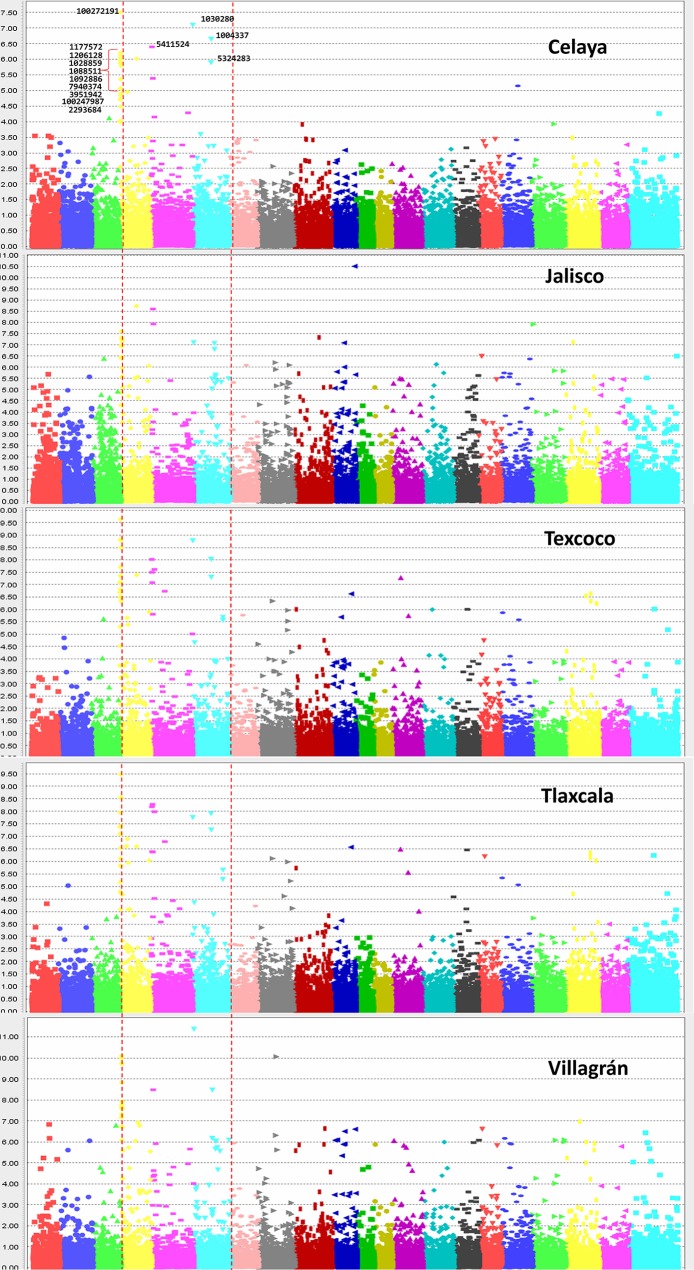
Manhattan plots showing the association of SNPs in the 419 lines with yellow rust resistance. The highly significant and common SNPs across five locations are labeled only in the top plot.

**Figure 6:**
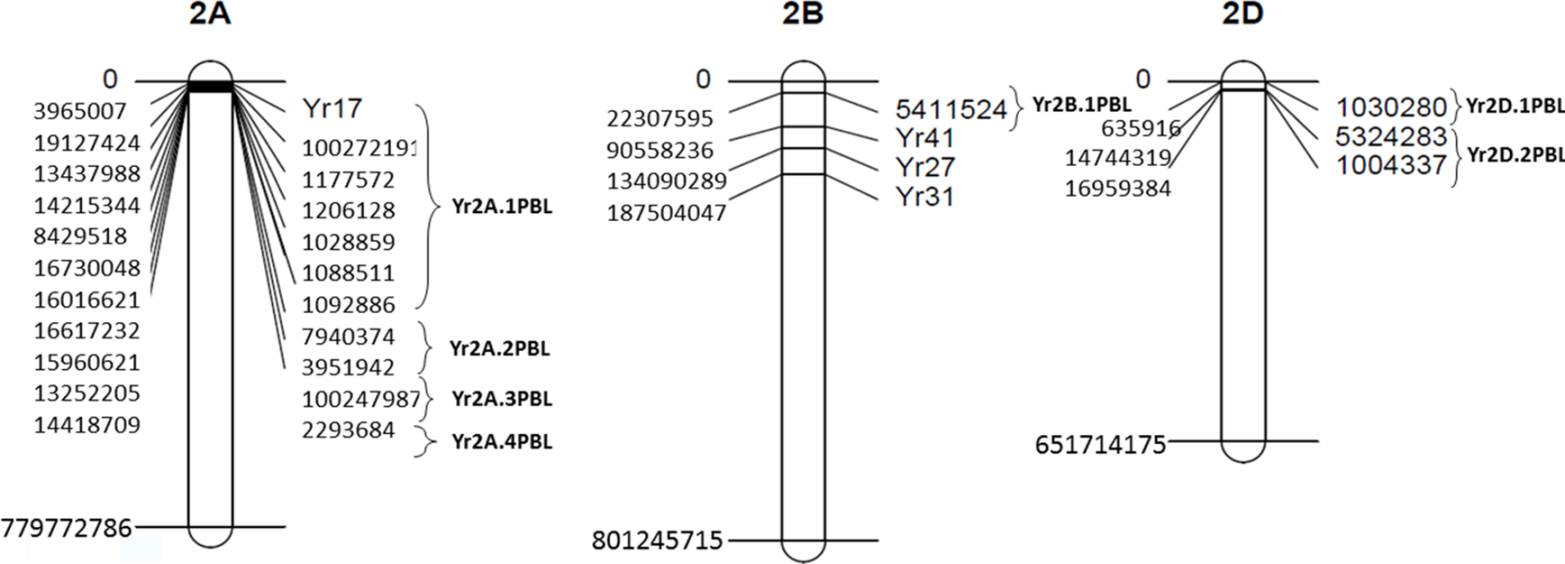
Map of yellow rust (YR) resistance genes and associated SNPs on chromosomes 2A, 2B, and 2D. Clone ID of SNPs and YR genes are indicated to the right side of the chromosomes while physical positions are shown to the left.

Across five locations, 14 SNPs (**Table 1**) from three chromosomes were significantly *P*-value <10^−6^ associated with YR severity (**Figures 5** and **6**) with phenotypic variation (*R*^2^) ranging from 6.0 to 14.2%. The following 10 SNPs from chromosome 2A were significantly associated; 100272191 and 1177572 at 8.29 cM, 1206128 at 8.6 cM, 1028859 and 1088511 at 9.93 cM, 1092886 at 9.97 cM, 7940374 at 11.57 cM, 3951942 at 13.34 cM, 100247987 at 17.84 cM, and 2293684 at 75.64 cM. Based on an average LD decay of 2.5 cM, these 10 marker–trait associations were classified into four QTLs (Yr2A.1PBL, Yr2A.2PBL, Yr2A.3PBL, and Yr2A.4PBL, **Table 1**). On chromosome 2B, SNP marker 5411524 at 8.94 cM (Yr2B.1PBL) was associated with YR resistance with percentage variation ranging from 7.4 to 10.2% in different environments. Three SNPs; 1030280 (0.65 cM), 1004337 (82.6 cM), and 5324283 (83.01cM) were classified into two QTL (Yr2D.1PBL and Yr2D.2PBL) on chromosome 2D, which explained 14.2, 14.1, and 11.1% of the phenotypic variation, respectively.

**Table 1:** QTL/SNPs associated with yellow rust resistance across five locations in Mexico.

QTL name	SNP	Chr	Pos (cM)	Physical position	Celaya			Jalisco			Texcoco			Tlaxcala			Villagrán		
					*P-value*	*FDR*	*R^2^ (%)*	*P-value*	*FDR*	*R^2^ (%)*	*P-value*	*FDR*	*R^2^ (%)*	*P-value*	*FDR*	*R^2^ (%)*	*P-value*	*FDR*	*R^2^ (%)*
Yr2A.1PBL	100272191|F|0-37:C > G-37:C > G	2A	8.29	19127424	1.05E−06	0.002	7.2	3.72E−07	0.001	7.5	8.38E−08	0.00181	8.2	1.45E−06	0.0088	6.8	2.03E−08	4.64E−05	9.9
	1177572|F|0-15:T > G-15:T > G	2A	8.29	13437988	3.16E−08	0.002	9.5	4.05E−07	0.062	7.9	7.82E−08	0.00912	8.9	4.28E−08	0.0007	8.9	7.95E−11	4.64E−05	13
	1206128|F|0-54:G > A-54:G > A	2A	8.6	14215344	8.52E−06	0.005	6	2.48E−08	0.001	8.9	1.53E−09	0.00003	10.3	1.11E−08	0.0001	9.2	4.75E−08	0.00022	9
	1028859|F|0-50:G > C-50:G > C	2A	9.93	8429518	9.63E−07	0.024	7.7	3.43E−07	0.009	8.5	1.57E−07	0.00166	9.3	8.34E−08	0.002	9.3	1.87E−07	0.00457	9.7
	1088511|F|0-7:T > C-7:T > C	2A	9.93	16730048	7.96E−07	0.005	7.1	1.03E−07	0.004	8.2	4.38E−08	0.00018	8.5	7.64E−07	0.0003	7	1.89E−10	3.26E−05	11.6
	1092886|F|0-39:A > C-39:A > C	2A	9.97	16016621	1.31E−06	0.005	7	3.62E−07	0.004	7.6	1.95E−08	0.00016	9	4.06E−08	0.0006	8.7	1.63E−08	0.00022	9.4

Yr2A.2PBL	7940374|F|0-14:A > C-14:A > C	2A	11.5	16617232	6.25E−07	0.002	7.7	4.68E−08	0.001	9	3.04E−07	0.0046	8.4	2.91E−07	0.0009	8.4	1.45E−09	1.13E−05	11.8
	3951942|F|0-8:A > G-8:A > G	2A	13.3	15960621	4.35E−06	0.066	6.6	5.32E−08	0.021	9.1	3.32E−07	0.05423	8.1	3.74E−08	0.001	8.9	2.48E−08	0.00322	9.8

Yr2A.3PBL	100247987|F|0-40:G > A-40:G > A	2A	17.8	13252205	1.61E−06	0.003	7	6.78E−08	0.003	8.5	4.61E−07	0.00083	7.4	1.55E−06	0.0034	6.8	1.16E−08	0.00022	10

Yr2A.4PBL	2293684|F|0-18:C > T-18:C > T	2A	75.6	14418709	9.59E−07	0.005	7.5	2.69E−06	0.03	7.1	4.06E−08	0.00106	9.5	2.51E−07	0.0033	8.1	1.15E−07	0.0014	9.3

Yr2B.1PBL	5411524|F|0-14:G > A-14:G > A	2B	8.94	22307595	4.01E−07	0.001	7.4	2.51E−09	0.001	10.1	9.59E−09	0.00018	9.1	6.53E−09	0.0001	9.4	3.23E−09	0.00018	10.2

Yr2D.1PBL	1030280|F|0-14:G > C-14:G > C	2D	0.65	635916	7.93E−08	0.001	8.4	7.85E−08	0.004	8.3	1.66E−09	0.00003	10.2	1.74E−08	0.0001	8.9	4.23E−12	3.09E−05	13.8

Yr2D.2PBL	1004337|F|0-6:C > T-6:C > T	2D	82.6	16959384	1.27E−06	0.001	7	9.17E−06	0.018	5.9	9.29E−09	0.00014	9.4	1.22E−08	0.0001	9.3	6.63E−07	0.00197	7.8
	5324283|F|0-29:A > G-29:A > G	2D	83.0	14744319	2.25E−07	0.006	8.5	3.25E−06	0.126	7.7	4.97E−08	0.00194	9.6	5.46E−08	0.0012	9.7	3.28E−09	0.00201	11.8

Chr, chromosome; Pos, position in cM.

The analysis by location showed variable *R*^2^ as well as level of significance of 14 SNPs. Jalisco was the locality where the markers explained lower percentage of the phenotypic variation for YR resistance with values of 5.9 to 9%; Villagrán, on the other hand, showed the highest values with a range of 7.8 to 13.8% (**Table 1**). When comparing some of the best lines (0 to 5% severity) with some of the worst (36 to 54% severity) for their response to rust, it was observed that the resistant ones carried practically all 14 resistant alleles, however, the susceptible lines had very few resistant alleles (**Supplementary Tables 4** and **5**). A general trend that emerged was that the more resistant alleles the lines had, the greater was their resistance, suggesting an additive effect of the loci involved.

**Figure 7:**
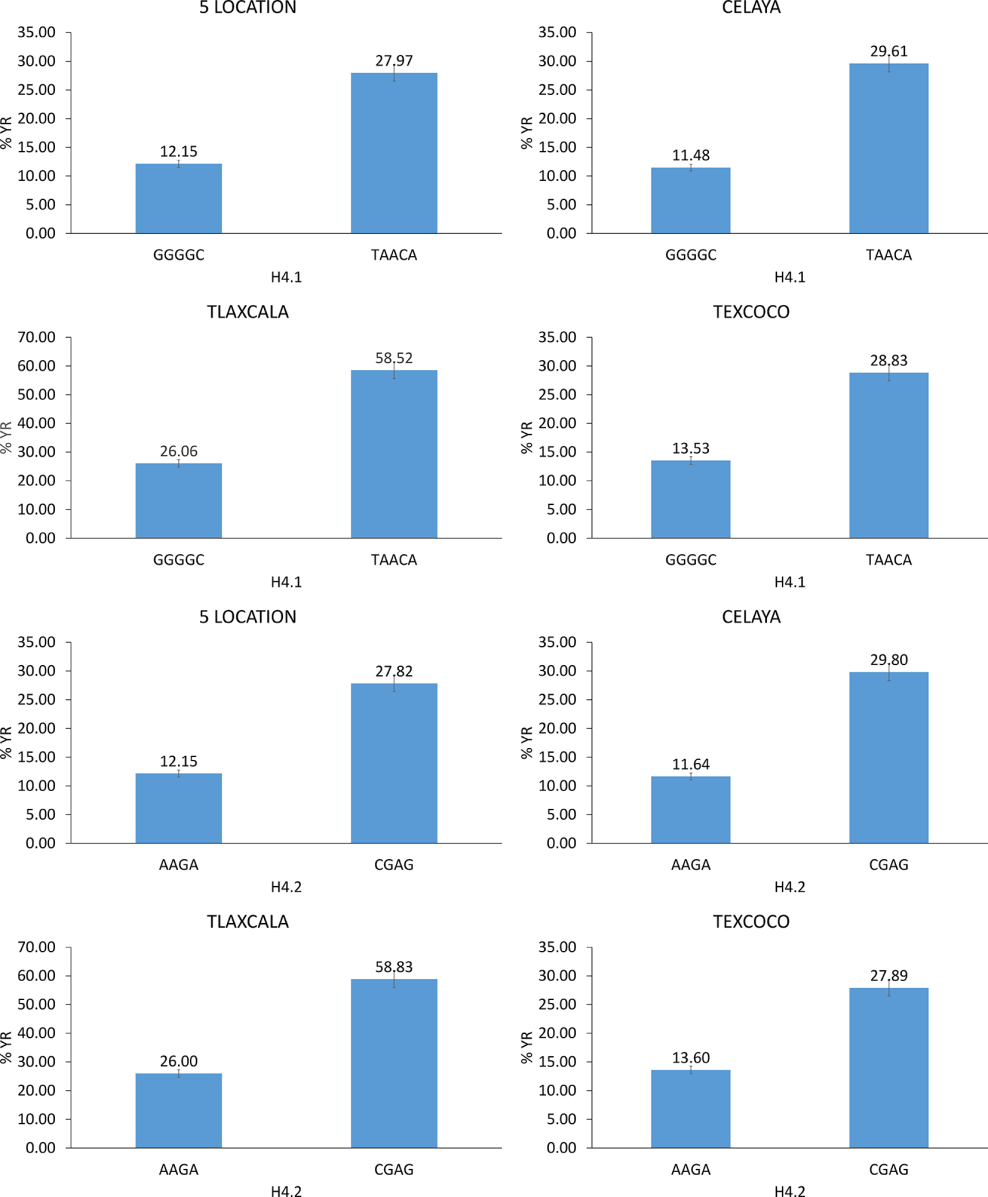
Allelic effects of significant haplotypes associated with YR resistance.

Out of 591 haplotype blocks obtained genome-wide, seven showed significant association (*P*-value <10^−6^) with YR resistance (**Table 2**) and were located on chromosomes 1B (one), 2A (five), and 2B (one). The haplotype block on chromosome 1B, H2.5, was detected in Jalisco. The haplotype blocks H4.1, H4.2, and H4.18 on chromosome 2A were found to be significant in five locations, while blocks H4.4 and H4.5 were significant in one or two locations. The block H5.20 on chromosome 2B was found to be significant only in the state of Jalisco. Of the seven haplotype blocks, five are made up of two SNPs (H2.5, H4.18, H4.4, H4.5, and H5.20) and remaining two (H4.1 and H4.2) with five and four SNPs, respectively. The allelic effect of these haplotypes on percentage of severity of YR ranged between 7.9 and 19.9% among locations and 8.1 and 24.0% across locations. Of the 14 significant SNPs identified in this study, seven were contained in two significant haplotypes. The SNPs *1177572*, *1206128*, *1028859*, *1092886*, and *5411524* were part of haplotype block H4.1 and SNPs *7940774* and *3951942* were part of the block H4.2. The allelic combination in the associated haplotype blocks that showed a difference in the percentage of YR severity greater than or equal to 15% was considered as important. With H4.1, differences of 18.13% in Celaya, 32.45% in Tlaxcala, 15.30% in Texcoco, and 15.82% in the average of the five locations (**Figure 7**) were detected between the favorable and unfavorable alleles. The allelic combination that showed the lowest percentage of rust was GGGGC. Haplotype H4.2 detected differences in Celaya of 18.17%, in Tlaxcala of 32.83%, in Texcoco of 14.29%, and in the average of the five locations of 15.67%. The allelic combination that showed lower percentage of rust was AAGA.

**Table 2:** Identified significant haplotypes associated with yellow resistance across five locations.

Haplotype	Chromosome	SNPs	Location	*P-value*	*R^2^ (%)*
H2.5	1B	1096067|F|0-12:G > A-12:G > A	Jalisco	1.77E−06	8.752
		1082843|F|0-43:T > C-43:T > C			
H4.1	2A	1177572|F|0-15:T > G-15:T > G	Villagrán	4.72E−14	19.898
		1206128|F|0-54:G > A-54:G > A	Texcoco	1.42E−15	17.884
		5411524|F|0-14:G > A-14:G > A	Tlaxcala	6.56E−21	19.948
		1028859|F|0-50:G > C-50:G > C	Jalisco	5.71E−08	12.839
		1092886|F|0-39:A > C-39:A > C	Celaya	1.95E−14	18.113
H4.2	2A	7940374|F|0-14:A > C-14:A > C	Villagrán	4.73E−14	19.897
		1262031|F|0-29:G > A-29:G > A	Texcoco	3.38E−14	16.773
		3951942|F|0-8:A > G-8:A > G	Tlaxcala	1.58E−20	19.695
		100280868|F|0-13:G > A-13:G > A	Jalisco	1.42E−10	15.568
			Celaya	1.57E−15	19.053
H4.4	2A	1277633|F|0-33:G > A-33:G > A	Villagrán	2.95E−06	7.999
		2258664|F|0-28:A > G-28:A > G			
H4.5	2A	3064800|F|0-41:C > T-41:C > T	Tlaxcala	2.11E−09	9.407
H4.18	2A	100278258|F|0-45:G > C-45:G > C	Villagrán	1.53E−12	14.445
		1287063|F|0-34:G > A-34:G > A	Texcoco	2.30E−19	17.339
			Tlaxcala	1.72E−25	19.69
			Jalisco	5.63E−07	8.359
			Celaya	2.14E−14	14.485

H5.20	2B	1209087|F|0-29:C > G-29:C > G	Jalisco	6.84E−07	8.741
		1101006|F|0-6:G > T-6:G > T			

*In silico* analysis of the 14-associated SNPs revealed hits with four candidate genes, which are related to pathogenic processes or known to regulate induction of genes related to pathogenesis (**Supplementary Table 6**).

## Discussion

The prevalence and severity of YR in the selected environments for this study presented variations: in environments with humid and fresh climate, there was higher severity, while in warmer and drier environments the severity was lower; on the other hand, rust races present naturally were not always the same. However, in all cases the behavior of wheat lines against rust infection maintained the same pattern *i.e.* lines with higher resistance or susceptibility were resistant or susceptible in almost all environments. Frequencies of lines in the histograms showed a skewed distribution to the left where the resistant lines are represented, which could be attributed to the selective pressure exerted by the breeders through directed crosses.

Population structure can play as a confounding factor in GWAS analysis and it should be dealt with to avoid false associations. STRUCTURE and PCA are two widely used tools to infer cryptic population structure from genome-wide data such as high density SNPs (Abraham and Inouye, 2014). Both analysis showed three subpopulations/subgroups (SP 1–SP 3) (**Figures 2** and **3**). SP1 grouped 131 lines that averaged the highest severity value of YR (21.6%) and a range of 4 to 54%. The lines of SP1 shared parents Baj#1, Reedling#1, Villa Juarez F2009, and Tacupeto F2001 in their pedigree. SP2 formed a group of 110 lines with an average severity of 14.8% and a range of 5.1 to 47.5%. Seri.1b//Kauz/Hevo/3/Amad*2/4/Kiritati was the common parent shared by 45.9% of the group. SP3 was the largest group with 178 lines. This group recorded the lowest average severity of YR (12.6%), with a range of 4 to 32%. This group had parent Fret2*2/4/Sni/Trap#1/3/Kauz*2/Trap//Kauz/5/Kachu common in pedigrees of most of the lines.

LD is reported to decay at short distance and hence faster in outcrossing crop species, such as maize (Dinesh et al., 2016), and at large distance (up to 40 cM) and hence slower in self-pollinated crop species, such as wheat (Yu et al., 2014). Extent of LD decay depends upon genetic distance and it determines the number of markers required for association mapping (Mather et al., 2007). LD decay varies widely among wheat populations, ranging from 5 or 10 cM (Würschum et al., 2013; Edae et al., 2014; Zegeye et al., 2014; Sehgal et al., 2017) to 20 or >20 cM (Crossa et al., 2007; Somers et al., 2007; Benson et al., 2012; Chen et al., 2012). In the present study, the extent of LD decay (*r*^2^ = 0.1) was 2.5 cM for whole genome (**Figure 4**), which suggests higher genetic diversity of the investigated pre-breeding panel as compared to previous studies This could be attributed to the way the panel has been generated. A three way crossing scheme (exotic/elite1//elite2) among exotics and elites was employed to generate PBLs in such a way that each PBL acquired approximately 25% of the exotic genome and 75% of the elite genome at an early stage (Singh et al., 2018). Therefore, exotic alleles were incorporated into elite backgrounds even before their trait values were identified. This strategy enabled investigation of greater number of genetic variants at a time and allowed recombination between exotic and elite genomes. In addition, many previous studies reported the slowest LD decay in D genome as compared to A and B genomes (Chao et al., 2007). In this study, a faster LD decay in the D genome was observed; comparable to A genome and faster than B genome (**Figure 4**), which could be attributed to the use of synthetics for developing PBLs, driving more recombination in the D genome.

Rosewarne et al. (2013) reported metaQTL genomic regions for YR resistance by using information of 140 QTL from over 60 publications. We compared the locations of the significant QTL identified in the present study with metaQTL in Rosewarne et al. (2013) and with latest studies (Naruoka et al., 2015; Bulli et al., 2016; Long et al., 2019). We also used interactive map containing information of all stripe rust genes in MASwheat (https://maswheat.ucdavis.edu/protocols/YellowRust/YellowRustMap.html). Four QTL were identified on chromosome 2A on short arm in the present study. Of these, Yr2A.1PBL and Yr2A.2PBL overlapped with the metaQTL QRYr2A.1 on 2AS (Rosewarne et al., 2013) where Yr56 and Yr17 are located. However, when we compared physical positions, Yr17 was 4 to 15 Mb away from markers in Yr2A.1PBL and Yr2A.2PBL. The two QTL reported recently by Long et al. (2019) and Bulli et al. (2016) on 2AS were also part of metaQTL QRYr2A.1. Of the remaining two QTL, Yr2A.4PBL is more towards distal end of 2AS where Long et al. (2019) reported a novel locus QDL.sicau-2A based on disease severity scores. Hence, it is highly likely that out of the four, Yr2A.3PBL is a novel locus. BLAST analysis of significant SNPs on 2A revealed hits with three candidate genes (**Supplementary Table 6**). The marker 1092886 showed similarity with glunolactone oxidase gene that regulates the final stage of the synthesis of ascorbic acid, which is related to the pathogenic processes and the activation of reactive oxygen species (Smirnoff, 1996). The marker 7940374 identified a gene that produces an acyl transferase, such as quinate O-hydroxycinnamoyl transferase, which regulates the production of secondary metabolites derived from the tyrosine and phenylalanine routes (Hirschmann et al., 2014). This is one of the essential enzymes for the synthesis of hydroxycinnamic acid amides (HCAAs), which are known to regulate fundamental processes such as the responses of plants to biotic and abiotic stress (Campos et al., 2014). It has been shown that the increase in HCAAs is accompanied by an increase in salicylic acid levels and induction of genes related to pathogenesis (Macoy et al., 2015). SNP 1028859 is related to functions of proteins with F-box and DUF domains, silencing of which in *Arabidopsis* has shown to confer drought tolerance mediated by ABA (Kim et al., 2012). The role of F-box proteins in pathogenic defense is still unknown.

On 2B, Yr2B.1PBL (identified by SNP 5411524) on short arm reduced YR severity by 43%. There are three metaQTL on 2BS, of which metaQTL QRYr2B.2 particularly is a gene rich region containing a number of seedling and APR genes, for example, *Yr27* and *Yr31*. The Yr2B.1PBL identified here is part of metaQTL QRYr2B.2; while the two QTL reported by Long et al. (2019) were part of metaQTL QRYr2B.3. Based on physical position, *Yr27* and *Yr31* are 53 to 111Mb away from SNP 5411524. *In silico* analysis of the SNP 5411524 showed candidate hits with TraesCS2B02G045300, a NB-ARC domain. The NB-ARC domain is a functional ATPase domain, and its nucleotide-binding state is proposed to regulate activity of the R protein. It is proposed that binding and hydrolysis of ATP by this domain induces conformational changes in the overall protein, leading to formation of the apoptosome, which leads to cell death. Many genes have been reported to contribute to wheat resistance against stripe rust fungus by regulating cell death. Studies of expression profiles of NB-ARC genes in wheat demonstrated their participation in response to leaf rust *P. triticina* (Chandra et al., 2017). Hence, it could be possible that this SNP is tightly linked to a seedling resistance gene. These results warrant an in-depth analysis (allelism test) to determine whether it is a novel gene or an allele of one of previously mapped genes on 2BS.

There are three metaQTL regions associated with YR resistance on chromosome 2D. The one reported on 2DS was identified in a single study (Lu et al., 2009). Naruoka et al. (2015) recently reported a new stable APR locus on 2DS (stable in nine environments) in a winter wheat association panel. Here, we identified two QTL, Yr2D.1PBL and Yr2D.2PBL, both on short arm of chromosome 2D. These represent potentially novel resistance loci as confidence interval of these QTL tagged by SNPs 1030280, 1004337, and 5324283 did not overlap with the position of previously published QTL on 2DS. The genes *Yr16* and *Yr54* are located on 2DL. Lines having Yr2D.2PBL had on an average 9.2% of rust severity against 16.25% of those that do not have it, indicating the effectiveness of this new QTL. BLAST analysis of SNP 1030280 identified a gene that produces the two-component histidine kinase related to the ethylene response sensor 2, which elicits the response to ethylene. This alkene plays a very important role in the final regulation of multiple metabolic processes of plants, including the induction of defense mechanisms mediated by jasmonate (Lorenzo et al., 2003; McGrath et al., 2005; Pré et al., 2008; Wasternack, 2015).

Use of multi-allelic haplotypes has significantly improved the power and robustness of GWAS studies in major crops including soybean (Hao et al., 2012), barley (Lorenz et al., 2010), and maize (Lu et al., 2012). A recent study in durum wheat also showed that the haplotype-based analysis resulted in an increase of the phenotypic variance explained (50.4% on average) and the allelic effect (33.7% on average) when compared to single marker analysis (N’Diaye et al., 2017). When we compared the results obtained by haplotype-based GWAS with SNP-based GWAS, we found that the percentage variation (*R*^2^) explained by haplotypes ranged from 7.9 to 19.9% in different locations, while for the associated 14 significant SNPs it ranged from 6.0 to 14%, resulting in an average 6.5% higher *R*^2^ by haplotypes compared to single SNPs. This clearly demonstrates the advantage provided by the former approach in identifying genomic regions that control YR resistance.

## Conclusions

This research identified 14 SNP markers and 7 haplotypes associated with YR resistance in a diverse set of pre-breeding lines. These associations were delimited to seven QTLs based on average LD decay of 2.5 cM. Three of these QTLs are likely to be novel and represent attractive targets for marker-assisted selection. *In silico* analysis of the SNPs revealed four candidate genes known to regulate induction of genes related to pathogenesis. In depth upstream analysis of these genes can help dissect the underlying mechanism of YR resistance in PBLs.

## Data Availability Statement

The datasets generated for this study can be found in the CIMMYT Research Data & Software Repository Network https://data.cimmyt.org/dataset.xhtml?persistentId=hdl:11529/10548313.

## Author Contributions

Conceived and designed the study: SS, ES-M. Performed the study: LL-R, ES-M. Analyzed the data: DS, LL-R, SS, ES-M, GI. Contributed materials: MR-V, VM-T, CS, JB, CO, CA-M, JR-P, PV. Genotyping/analysis tools: SS, PV, ES-M, CS, DS. Interpretation of results: LL-R, DS, JB, SS, ES-M, VM-T. Wrote first draft of the paper: LL-R, DS, SS, ES-M. Revised the manuscript: DS, LL-R, SS, DS, ES-M, GI, MR-V, VM-T, CS, JB, CO, CA-M, JR-P, PV.

## Conflict of Interest

The authors declare that the research was conducted in the absence of any commercial or financial relationships that could be construed as a potential conflict of interest.
