# Supplementary material for: GWAS to Identify Genetic Loci for Resistance to Yellow Rust in Wheat Pre-Breeding Lines Derived From Diverse Exotic Crosses
Source: Front Plant Sci. 2019 Oct 30;10:1390. doi: 10.3389/fpls.2019.01390 (PMC6831551; doi:10.3389/fpls.2019.01390)
Supplement: Supplementary file 1 [file Table_1.docx]

**SUPPLEMENTARY MATERIAL**

| **Table Sup. 1**. Lines phenotyped for yellow rust in Celaya, Villagrán (Guanajuato) and La Barca (Jalisco) in the Fall Winter (FW) cycle 2015-16 and in Texcoco, State of Mexico and Nanacamilpa, Tlaxcala in the Spring Summer (SS) 2016 cycle. | | | |
| --- | --- | --- | --- |
| **#** | **GID** | **LN** | **PEDIGREE** |
| 1 | 7640765 | LTP | CETA/AE.SQUARROSA (665)//KACHU/3/BAJ #1 |
| 2 | 7640767 | LTP | CETA/AE.SQUARROSA (665)//KACHU/3/BAJ #1 |
| 3 | 7640781 | LTP | CROC_1/AE.SQUARROSA (466)//KACHU/3/BAJ #1 |
| 4 | 7640788 | LTP | CROC_1/AE.SQUARROSA (466)//KACHU/3/BAJ #1 |
| 5 | 7640794 | LTP | CROC_1/AE.SQUARROSA (466)//KACHU/3/BAJ #1 |
| 6 | 7640798 | LTP | CROC_1/AE.SQUARROSA (466)//KACHU/3/BAJ #1 |
| 7 | 7640814 | LTP | CROC_1/AE.SQUARROSA (481)//KACHU/3/BAJ #1 |
| 8 | 7640824 | LTP | KE90-282/MILAN//KACHU/3/BAJ #1 |
| 9 | 7640833 | LTP | KE90-282/MILAN//KACHU/3/BAJ #1 |
| 10 | 7640845 | LTP | KE90-282/MILAN//KACHU/3/BAJ #1 |
| 11 | 7640848 | LTP | KE90-282/MILAN//KACHU/3/BAJ #1 |
| 12 | 7640852 | LTP | KE90-282/MILAN//KACHU/3/BAJ #1 |
| 13 | 7640857 | LTP | KE90-282/MILAN//KACHU/3/BAJ #1 |
| 14 | 7640860 | LTP | KE90-282/MILAN//KACHU/3/BAJ #1 |
| 15 | 7640864 | LTP | KE90-282/MILAN//KACHU/3/BAJ #1 |
| 16 | 7640926 | LTP | IG 41485/KACHU//BAJ #1 |
| 17 | 7640950 | LTP | IG 42158/KACHU//BAJ #1 |
| 18 | 7640952 | LTP | IG 42158/KACHU//BAJ #1 |
| 19 | 7640955 | LTP | SCOOP_1/AE.SQUARROSA (634)//KACHU/3/BAJ #1 |
| 20 | 7640964 | LTP | SCOOP_1/AE.SQUARROSA (634)//KACHU/3/BAJ #1 |
| 21 | 7640980 | LTP | SCOOP_1/AE.SQUARROSA (634)//KACHU/3/BAJ #1 |
| 22 | 7641281 | LTP | GAN/AE.SQUARROSA (897)//KACHU/3/BAJ #1 |
| 23 | 7641305 | LTP | CROC_1/AE.SQUARROSA (516)//KACHU/3/BAJ #1 |
| 24 | 7641307 | LTP | CROC_1/AE.SQUARROSA (516)//KACHU/3/BAJ #1 |
| 25 | 7641340 | LTP | CROC_1/AE.SQUARROSA (517)//KACHU/3/BAJ #1 |
| 26 | 7641345 | LTP | CROC_1/AE.SQUARROSA (517)//KACHU/3/BAJ #1 |
| 27 | 7641346 | LTP | CROC_1/AE.SQUARROSA (517)//KACHU/3/BAJ #1 |
| 28 | 7641347 | LTP | CROC_1/AE.SQUARROSA (517)//KACHU/3/BAJ #1 |
| 29 | 7641373 | LTP | CROC_1/AE.SQUARROSA (517)//KACHU/3/BAJ #1 |
| 30 | 7641375 | LTP | CROC_1/AE.SQUARROSA (517)//KACHU/3/BAJ #1 |
| 31 | 7641379 | LTP | CROC_1/AE.SQUARROSA (517)//KACHU/3/BAJ #1 |
| 32 | 7641390 | LTP | DOY1/AE.SQUARROSA (1024)//KACHU/3/BAJ #1 |
| 33 | 7641406 | LTP | DOY1/AE.SQUARROSA (1024)//KACHU/3/BAJ #1 |
| 34 | 7641414 | LTP | DOY1/AE.SQUARROSA (1024)//KACHU/3/BAJ #1 |
| 35 | 7641424 | LTP | CETA/AE.SQUARROSA (1025)//KACHU/3/BAJ #1 |
| 36 | 7641427 | LTP | CETA/AE.SQUARROSA (1025)//KACHU/3/BAJ #1 |
| 37 | 7641438 | LTP | CETA/AE.SQUARROSA (1025)//KACHU/3/BAJ #1 |
| 38 | 7641441 | LTP | CETA/AE.SQUARROSA (1025)//KACHU/3/BAJ #1 |
| 39 | 7641448 | LTP | CETA/AE.SQUARROSA (1025)//KACHU/3/BAJ #1 |
| 40 | 7641450 | LTP | CETA/AE.SQUARROSA (1025)//KACHU/3/BAJ #1 |
| 41 | 7641452 | LTP | CETA/AE.SQUARROSA (1025)//KACHU/3/BAJ #1 |
| 42 | 7641453 | LTP | ARLIN_1/AE.SQUARROSA (1017)//KACHU/3/BAJ #1 |
| 43 | 7641502 | LTP | CETA/AE.SQUARROSA (1055)//KACHU/3/BAJ #1 |
| 44 | 7641513 | LTP | CETA/AE.SQUARROSA (1055)//KACHU/3/BAJ #1 |
| 45 | 7641559 | LTP | D67.2/PARANA 66.270//AE.SQUARROSA (185)/3/KACHU/4/BAJ #1 |
| 46 | 7641562 | LTP | D67.2/PARANA 66.270//AE.SQUARROSA (185)/3/KACHU/4/BAJ #1 |
| 47 | 7641563 | LTP | D67.2/PARANA 66.270//AE.SQUARROSA (185)/3/KACHU/4/BAJ #1 |
| 48 | 7641642 | LTP | GAN/AE.SQUARROSA (680)//KACHU/3/BAJ #1 |
| 49 | 7641656 | LTP | CETA/AE.SQUARROSA (681)//KACHU/3/BAJ #1 |
| 50 | 7641672 | LTP | CETA/AE.SQUARROSA (681)//KACHU/3/BAJ #1 |
| 51 | 7641675 | LTP | CROC_1/AE.SQUARROSA (298)//KACHU/3/BAJ #1 |
| 52 | 7641701 | LTP | CROC_1/AE.SQUARROSA (298)//KACHU/3/BAJ #1 |
| 53 | 7641708 | LTP | CROC_1/AE.SQUARROSA (298)//KACHU/3/BAJ #1 |
| 54 | 7641715 | LTP | CROC_1/AE.SQUARROSA (298)//KACHU/3/BAJ #1 |
| 55 | 7641726 | LTP | CROC_1/AE.SQUARROSA (298)//KACHU/3/BAJ #1 |
| 56 | 7641729 | LTP | CROC_1/AE.SQUARROSA (298)//KACHU/3/BAJ #1 |
| 57 | 7641741 | LTP | DVERD_2/T.URARTU (545)//KACHU/3/BAJ #1 |
| 58 | 7641769 | LTP | IG 42153/KACHU//BAJ #1 |
| 59 | 7641771 | LTP | IG 42153/KACHU//BAJ #1 |
| 60 | 7641781 | LTP | IG 42153/KACHU//BAJ #1 |
| 61 | 7641789 | LTP | IG 42153/KACHU//BAJ #1 |
| 62 | 7641791 | LTP | IG 42153/KACHU//BAJ #1 |
| 63 | 7641796 | LTP | H-1442/KACHU//BAJ #1 |
| 64 | 7641814 | LTP | H-1442/KACHU//BAJ #1 |
| 65 | 7641825 | LTP | H-1442/KACHU//BAJ #1 |
| 66 | 7641834 | LTP | H-1442/KACHU//BAJ #1 |
| 67 | 7641835 | LTP | H-1442/KACHU//BAJ #1 |
| 68 | 7641845 | LTP | W47/KACHU//BAJ #1 |
| 69 | 7641858 | LTP | W47/KACHU//BAJ #1 |
| 70 | 7641901 | LTP | CHEN/AE.SQ//2*OPATA/3/BAJ #1/4/SUP152 |
| 71 | 7641904 | LTP | CHEN/AE.SQ//2*OPATA/3/BAJ #1/4/SUP152 |
| 72 | 7641906 | LTP | CHEN/AE.SQ//2*OPATA/3/BAJ #1/4/SUP152 |
| 73 | 7641907 | LTP | CHEN/AE.SQ//2*OPATA/3/BAJ #1/4/SUP152 |
| 74 | 7641908 | LTP | CHEN/AE.SQ//2*OPATA/3/BAJ #1/4/SUP152 |
| 75 | 7641909 | LTP | CHEN/AE.SQ//2*OPATA/3/BAJ #1/4/SUP152 |
| 76 | 7641911 | LTP | CHEN/AE.SQ//2*OPATA/3/BAJ #1/4/SUP152 |
| 77 | 7641915 | LTP | CHEN/AE.SQ//2*OPATA/3/BAJ #1/4/SUP152 |
| 78 | 7641917 | LTP | CHEN/AE.SQ//2*OPATA/3/BAJ #1/4/SUP152 |
| 79 | 7641924 | LTP | CHEN/AE.SQ//2*OPATA/3/BAJ #1/4/SUP152 |
| 80 | 7641926 | LTP | CHEN/AE.SQ//2*OPATA/3/BAJ #1/4/SUP152 |
| 81 | 7641934 | LTP | CHEN/AE.SQ//2*OPATA/3/BAJ #1/4/SUP152 |
| 82 | 7641939 | LTP | CHEN/AE.SQ//2*OPATA/3/BAJ #1/4/SUP152 |
| 83 | 7641941 | LTP | CHEN/AE.SQ//2*OPATA/3/BAJ #1/4/SUP152 |
| 84 | 7641951 | LTP | CHEN/AE.SQ//2*OPATA/3/BAJ #1/4/SUP152 |
| 85 | 7641960 | LTP | CHEN/AE.SQ//2*OPATA/3/BAJ #1/4/SUP152 |
| 86 | 7641971 | LTP | CHEN/AE.SQ//2*OPATA/3/BAJ #1/4/SUP152 |
| 87 | 7641975 | LTP | CHEN/AE.SQ//2*OPATA/3/BAJ #1/4/SUP152 |
| 88 | 7642063 | LTP | CETA/AE.SQUARROSA (166)//BAJ #1/3/SUP152 |
| 89 | 7642077 | LTP | D67.2/PARANA 66.270//AE.SQUARROSA (320)/3/BAJ #1/4/SUP152 |
| 90 | 7642204 | LTP | IRAQ-27/BAJ #1//SUP152 |
| 91 | 7642238 | LTP | IRAQ-31/BAJ #1//SUP152 |
| 92 | 7642281 | LTP | BCN/4/RABI//GS/CRA/3/AE.SQUARROSA (895)/5/SUP152/6/VILLA JUAREZ F2009 |
| 93 | 7642292 | LTP | BCN/4/RABI//GS/CRA/3/AE.SQUARROSA (895)/5/SUP152/6/VILLA JUAREZ F2009 |
| 94 | 7642308 | LTP | BCN/4/RABI//GS/CRA/3/AE.SQUARROSA (895)/5/SUP152/6/VILLA JUAREZ F2009 |
| 95 | 7642324 | LTP | BCN/4/RABI//GS/CRA/3/AE.SQUARROSA (895)/5/SUP152/6/VILLA JUAREZ F2009 |
| 96 | 7642370 | LTP | SORA/AE.SQUARROSA (442)//SUP152/3/VILLA JUAREZ F2009 |
| 97 | 7642372 | LTP | SORA/AE.SQUARROSA (442)//SUP152/3/VILLA JUAREZ F2009 |
| 98 | 7642377 | LTP | SORA/AE.SQUARROSA (442)//SUP152/3/VILLA JUAREZ F2009 |
| 99 | 7642378 | LTP | SORA/AE.SQUARROSA (442)//SUP152/3/VILLA JUAREZ F2009 |
| 100 | 7642412 | LTP | IG 122628/SUP152//VILLA JUAREZ F2009 |
| 101 | 7642418 | LTP | IG 122628/SUP152//VILLA JUAREZ F2009 |
| 102 | 7642443 | LTP | BCN//SORA/AE.SQUARROSA (323)/3/VILLA JUAREZ F2009/4/WBLL1/KUKUNA//TACUPETO F2001/3/BAJ #1 |
| 103 | 7642486 | LTP | BCN//CETA/AE.SEARSII (34D)/3/VILLA JUAREZ F2009/4/WBLL1/KUKUNA//TACUPETO F2001/3/BAJ #1 |
| 104 | 7642491 | LTP | CHEN/AE.SQ//WEAVER/3/VILLA JUAREZ F2009/4/WBLL1/KUKUNA//TACUPETO F2001/3/BAJ #1 |
| 105 | 7642492 | LTP | CHEN/AE.SQ//WEAVER/3/VILLA JUAREZ F2009/4/WBLL1/KUKUNA//TACUPETO F2001/3/BAJ #1 |
| 106 | 7642498 | LTP | CHEN/AE.SQ//WEAVER/3/VILLA JUAREZ F2009/4/WBLL1/KUKUNA//TACUPETO F2001/3/BAJ #1 |
| 107 | 7642500 | LTP | CHEN/AE.SQ//WEAVER/3/VILLA JUAREZ F2009/4/WBLL1/KUKUNA//TACUPETO F2001/3/BAJ #1 |
| 108 | 7642516 | LTP | CHEN/AE.SQ//WEAVER/3/VILLA JUAREZ F2009/4/WBLL1/KUKUNA//TACUPETO F2001/3/BAJ #1 |
| 109 | 7642579 | LTP | IG 42157/VILLA JUAREZ F2009/4/WBLL1/KUKUNA//TACUPETO F2001/3/BAJ #1 |
| 110 | 7642587 | LTP | IG 42157/VILLA JUAREZ F2009/4/WBLL1/KUKUNA//TACUPETO F2001/3/BAJ #1 |
| 111 | 7642590 | LTP | IG 42157/VILLA JUAREZ F2009/4/WBLL1/KUKUNA//TACUPETO F2001/3/BAJ #1 |
| 112 | 7642611 | LTP | IG 122793/VILLA JUAREZ F2009/4/WBLL1/KUKUNA//TACUPETO F2001/3/BAJ #1 |
| 113 | 7642612 | LTP | IG 122793/VILLA JUAREZ F2009/4/WBLL1/KUKUNA//TACUPETO F2001/3/BAJ #1 |
| 114 | 7642630 | LTP | IG 122793/VILLA JUAREZ F2009/4/WBLL1/KUKUNA//TACUPETO F2001/3/BAJ #1 |
| 115 | 7642634 | LTP | IG 126482/VILLA JUAREZ F2009/4/WBLL1/KUKUNA//TACUPETO F2001/3/BAJ #1 |
| 116 | 7642641 | LTP | IG 126482/VILLA JUAREZ F2009/4/WBLL1/KUKUNA//TACUPETO F2001/3/BAJ #1 |
| 117 | 7642643 | LTP | IG 126482/VILLA JUAREZ F2009/4/WBLL1/KUKUNA//TACUPETO F2001/3/BAJ #1 |
| 118 | 7642655 | LTP | BCN//CETA/AE.SEARSII (34D)/4/WBLL1/KUKUNA//TACUPETO F2001/3/BAJ #1/5/SERI.1B//KAUZ/HEVO/3/AMAD*2/4/KIRITATI |
| 119 | 7642657 | LTP | BCN//CETA/AE.SEARSII (34D)/4/WBLL1/KUKUNA//TACUPETO F2001/3/BAJ #1/5/SERI.1B//KAUZ/HEVO/3/AMAD*2/4/KIRITATI |
| 120 | 7642658 | LTP | BCN//CETA/AE.SEARSII (34D)/4/WBLL1/KUKUNA//TACUPETO F2001/3/BAJ #1/5/SERI.1B//KAUZ/HEVO/3/AMAD*2/4/KIRITATI |
| 121 | 7642659 | LTP | BCN//CETA/AE.SEARSII (34D)/4/WBLL1/KUKUNA//TACUPETO F2001/3/BAJ #1/5/SERI.1B//KAUZ/HEVO/3/AMAD*2/4/KIRITATI |
| 122 | 7642661 | LTP | BCN//CETA/AE.SEARSII (34D)/4/WBLL1/KUKUNA//TACUPETO F2001/3/BAJ #1/5/SERI.1B//KAUZ/HEVO/3/AMAD*2/4/KIRITATI |
| 123 | 7642662 | LTP | BCN//CETA/AE.SEARSII (34D)/4/WBLL1/KUKUNA//TACUPETO F2001/3/BAJ #1/5/SERI.1B//KAUZ/HEVO/3/AMAD*2/4/KIRITATI |
| 124 | 7642673 | LTP | BCN//CETA/AE.SEARSII (34D)/4/WBLL1/KUKUNA//TACUPETO F2001/3/BAJ #1/5/SERI.1B//KAUZ/HEVO/3/AMAD*2/4/KIRITATI |
| 125 | 7642683 | LTP | BCN//SORA/AE.SQUARROSA (323)/4/WBLL1/KUKUNA//TACUPETO F2001/3/BAJ #1/5/SERI.1B//KAUZ/HEVO/3/AMAD*2/4/KIRITATI |
| 126 | 7642685 | LTP | BCN//SORA/AE.SQUARROSA (323)/4/WBLL1/KUKUNA//TACUPETO F2001/3/BAJ #1/5/SERI.1B//KAUZ/HEVO/3/AMAD*2/4/KIRITATI |
| 127 | 7642686 | LTP | BCN//SORA/AE.SQUARROSA (323)/4/WBLL1/KUKUNA//TACUPETO F2001/3/BAJ #1/5/SERI.1B//KAUZ/HEVO/3/AMAD*2/4/KIRITATI |
| 128 | 7642689 | LTP | BCN//SORA/AE.SQUARROSA (323)/4/WBLL1/KUKUNA//TACUPETO F2001/3/BAJ #1/5/SERI.1B//KAUZ/HEVO/3/AMAD*2/4/KIRITATI |
| 129 | 7642691 | LTP | BCN//SORA/AE.SQUARROSA (323)/4/WBLL1/KUKUNA//TACUPETO F2001/3/BAJ #1/5/SERI.1B//KAUZ/HEVO/3/AMAD*2/4/KIRITATI |
| 130 | 7642693 | LTP | BCN//SORA/AE.SQUARROSA (323)/4/WBLL1/KUKUNA//TACUPETO F2001/3/BAJ #1/5/SERI.1B//KAUZ/HEVO/3/AMAD*2/4/KIRITATI |
| 131 | 7642694 | LTP | BCN//SORA/AE.SQUARROSA (323)/4/WBLL1/KUKUNA//TACUPETO F2001/3/BAJ #1/5/SERI.1B//KAUZ/HEVO/3/AMAD*2/4/KIRITATI |
| 132 | 7642695 | LTP | BCN//SORA/AE.SQUARROSA (323)/4/WBLL1/KUKUNA//TACUPETO F2001/3/BAJ #1/5/SERI.1B//KAUZ/HEVO/3/AMAD*2/4/KIRITATI |
| 133 | 7642702 | LTP | BCN//SORA/AE.SQUARROSA (323)/4/WBLL1/KUKUNA//TACUPETO F2001/3/BAJ #1/5/SERI.1B//KAUZ/HEVO/3/AMAD*2/4/KIRITATI |
| 134 | 7642704 | LTP | BCN//SORA/AE.SQUARROSA (323)/4/WBLL1/KUKUNA//TACUPETO F2001/3/BAJ #1/5/SERI.1B//KAUZ/HEVO/3/AMAD*2/4/KIRITATI |
| 135 | 7642705 | LTP | BCN//SORA/AE.SQUARROSA (323)/4/WBLL1/KUKUNA//TACUPETO F2001/3/BAJ #1/5/SERI.1B//KAUZ/HEVO/3/AMAD*2/4/KIRITATI |
| 136 | 7642707 | LTP | BCN//SORA/AE.SQUARROSA (323)/4/WBLL1/KUKUNA//TACUPETO F2001/3/BAJ #1/5/SERI.1B//KAUZ/HEVO/3/AMAD*2/4/KIRITATI |
| 137 | 7642709 | LTP | BCN//SORA/AE.SQUARROSA (323)/4/WBLL1/KUKUNA//TACUPETO F2001/3/BAJ #1/5/SERI.1B//KAUZ/HEVO/3/AMAD*2/4/KIRITATI |
| 138 | 7642710 | LTP | BCN//SORA/AE.SQUARROSA (323)/4/WBLL1/KUKUNA//TACUPETO F2001/3/BAJ #1/5/SERI.1B//KAUZ/HEVO/3/AMAD*2/4/KIRITATI |
| 139 | 7642712 | LTP | BCN//SORA/AE.SQUARROSA (323)/4/WBLL1/KUKUNA//TACUPETO F2001/3/BAJ #1/5/SERI.1B//KAUZ/HEVO/3/AMAD*2/4/KIRITATI |
| 140 | 7642713 | LTP | BCN//SORA/AE.SQUARROSA (323)/4/WBLL1/KUKUNA//TACUPETO F2001/3/BAJ #1/5/SERI.1B//KAUZ/HEVO/3/AMAD*2/4/KIRITATI |
| 141 | 7642716 | LTP | BCN//SORA/AE.SQUARROSA (323)/4/WBLL1/KUKUNA//TACUPETO F2001/3/BAJ #1/5/SERI.1B//KAUZ/HEVO/3/AMAD*2/4/KIRITATI |
| 142 | 7642718 | LTP | BCN//SORA/AE.SQUARROSA (323)/4/WBLL1/KUKUNA//TACUPETO F2001/3/BAJ #1/5/SERI.1B//KAUZ/HEVO/3/AMAD*2/4/KIRITATI |
| 143 | 7642723 | LTP | BCN//SORA/AE.SQUARROSA (323)/4/WBLL1/KUKUNA//TACUPETO F2001/3/BAJ #1/5/SERI.1B//KAUZ/HEVO/3/AMAD*2/4/KIRITATI |
| 144 | 7642733 | LTP | CHEN/AE.SQ//2*OPATA/4/WBLL1/KUKUNA//TACUPETO F2001/3/BAJ #1/5/SERI.1B//KAUZ/HEVO/3/AMAD*2/4/KIRITATI |
| 145 | 7642734 | LTP | CHEN/AE.SQ//2*OPATA/4/WBLL1/KUKUNA//TACUPETO F2001/3/BAJ #1/5/SERI.1B//KAUZ/HEVO/3/AMAD*2/4/KIRITATI |
| 146 | 7642740 | LTP | CHEN/AE.SQ//2*OPATA/4/WBLL1/KUKUNA//TACUPETO F2001/3/BAJ #1/5/SERI.1B//KAUZ/HEVO/3/AMAD*2/4/KIRITATI |
| 147 | 7642746 | LTP | CHEN/AE.SQ//2*OPATA/4/WBLL1/KUKUNA//TACUPETO F2001/3/BAJ #1/5/SERI.1B//KAUZ/HEVO/3/AMAD*2/4/KIRITATI |
| 148 | 7642748 | LTP | CHEN/AE.SQ//2*OPATA/4/WBLL1/KUKUNA//TACUPETO F2001/3/BAJ #1/5/SERI.1B//KAUZ/HEVO/3/AMAD*2/4/KIRITATI |
| 149 | 7642759 | LTP | CHEN/AE.SQ//2*OPATA/4/WBLL1/KUKUNA//TACUPETO F2001/3/BAJ #1/5/SERI.1B//KAUZ/HEVO/3/AMAD*2/4/KIRITATI |
| 150 | 7642761 | LTP | ARVAND 1/4/WBLL1/KUKUNA//TACUPETO F2001/3/BAJ #1/5/SERI.1B//KAUZ/HEVO/3/AMAD*2/4/KIRITATI |
| 151 | 7642763 | LTP | ARVAND 1/4/WBLL1/KUKUNA//TACUPETO F2001/3/BAJ #1/5/SERI.1B//KAUZ/HEVO/3/AMAD*2/4/KIRITATI |
| 152 | 7642767 | LTP | ARVAND 1/4/WBLL1/KUKUNA//TACUPETO F2001/3/BAJ #1/5/SERI.1B//KAUZ/HEVO/3/AMAD*2/4/KIRITATI |
| 153 | 7642770 | LTP | ARVAND 1/4/WBLL1/KUKUNA//TACUPETO F2001/3/BAJ #1/5/SERI.1B//KAUZ/HEVO/3/AMAD*2/4/KIRITATI |
| 154 | 7642777 | LTP | ARVAND 1/4/WBLL1/KUKUNA//TACUPETO F2001/3/BAJ #1/5/SERI.1B//KAUZ/HEVO/3/AMAD*2/4/KIRITATI |
| 155 | 7642781 | LTP | ARVAND 1/4/WBLL1/KUKUNA//TACUPETO F2001/3/BAJ #1/5/SERI.1B//KAUZ/HEVO/3/AMAD*2/4/KIRITATI |
| 156 | 7642785 | LTP | ARVAND 1/4/WBLL1/KUKUNA//TACUPETO F2001/3/BAJ #1/5/SERI.1B//KAUZ/HEVO/3/AMAD*2/4/KIRITATI |
| 157 | 7642786 | LTP | ARVAND 1/4/WBLL1/KUKUNA//TACUPETO F2001/3/BAJ #1/5/SERI.1B//KAUZ/HEVO/3/AMAD*2/4/KIRITATI |
| 158 | 7642792 | LTP | ARVAND 1/4/WBLL1/KUKUNA//TACUPETO F2001/3/BAJ #1/5/SERI.1B//KAUZ/HEVO/3/AMAD*2/4/KIRITATI |
| 159 | 7642809 | LTP | ARLIN_1/AE.SQUARROSA (536)/4/WBLL1/KUKUNA//TACUPETO F2001/3/BAJ #1/5/SERI.1B//KAUZ/HEVO/3/AMAD*2/4/KIRITATI |
| 160 | 7642833 | LTP | ARLIN_1/AE.SQUARROSA (536)/4/WBLL1/KUKUNA//TACUPETO F2001/3/BAJ #1/5/SERI.1B//KAUZ/HEVO/3/AMAD*2/4/KIRITATI |
| 161 | 7642850 | LTP | CETA/AE.SQUARROSA (1036)/4/WBLL1/KUKUNA//TACUPETO F2001/3/BAJ #1/5/SERI.1B//KAUZ/HEVO/3/AMAD*2/4/KIRITATI |
| 162 | 7642888 | LTP | CPI8/GEDIZ/3/GOO//ALB/CRA/4/AE.SQUARROSA (1038)/5/WBLL1/KUKUNA//TACUPETO F2001/3/BAJ #1/6/SERI.1B//KAUZ/HEVO/3/AMAD*2/4/KIRITATI |
| 163 | 7642906 | LTP | D67.2/PARANA 66.270//AE.SQUARROSA (828)/4/WBLL1/KUKUNA//TACUPETO F2001/3/BAJ #1/5/SERI.1B//KAUZ/HEVO/3/AMAD*2/4/KIRITATI |
| 164 | 7642911 | LTP | D67.2/PARANA 66.270//AE.SQUARROSA (828)/4/WBLL1/KUKUNA//TACUPETO F2001/3/BAJ #1/5/SERI.1B//KAUZ/HEVO/3/AMAD*2/4/KIRITATI |
| 165 | 7642917 | LTP | D67.2/PARANA 66.270//AE.SQUARROSA (828)/4/WBLL1/KUKUNA//TACUPETO F2001/3/BAJ #1/5/SERI.1B//KAUZ/HEVO/3/AMAD*2/4/KIRITATI |
| 166 | 7642918 | LTP | D67.2/PARANA 66.270//AE.SQUARROSA (828)/4/WBLL1/KUKUNA//TACUPETO F2001/3/BAJ #1/5/SERI.1B//KAUZ/HEVO/3/AMAD*2/4/KIRITATI |
| 167 | 7642935 | LTP | GAN/AE.SQUARROSA (741)/4/WBLL1/KUKUNA//TACUPETO F2001/3/BAJ #1/5/SERI.1B//KAUZ/HEVO/3/AMAD*2/4/KIRITATI |
| 168 | 7642936 | LTP | GAN/AE.SQUARROSA (741)/4/WBLL1/KUKUNA//TACUPETO F2001/3/BAJ #1/5/SERI.1B//KAUZ/HEVO/3/AMAD*2/4/KIRITATI |
| 169 | 7642939 | LTP | GAN/AE.SQUARROSA (741)/4/WBLL1/KUKUNA//TACUPETO F2001/3/BAJ #1/5/SERI.1B//KAUZ/HEVO/3/AMAD*2/4/KIRITATI |
| 170 | 7642941 | LTP | GAN/AE.SQUARROSA (741)/4/WBLL1/KUKUNA//TACUPETO F2001/3/BAJ #1/5/SERI.1B//KAUZ/HEVO/3/AMAD*2/4/KIRITATI |
| 171 | 7642958 | LTP | ALTAR 84/AE.SQUARROSA (1068)/4/WBLL1/KUKUNA//TACUPETO F2001/3/BAJ #1/5/SERI.1B//KAUZ/HEVO/3/AMAD*2/4/KIRITATI |
| 172 | 7642967 | LTP | 68.111/RGB-U//WARD RESEL/3/STIL/4/AE.SQUARROSA (188)/5/WBLL1/KUKUNA//TACUPETO F2001/3/BAJ #1/6/SERI.1B//KAUZ/HEVO/3/AMAD*2/4/KIRITATI |
| 173 | 7642968 | LTP | 68.111/RGB-U//WARD RESEL/3/STIL/4/AE.SQUARROSA (188)/5/WBLL1/KUKUNA//TACUPETO F2001/3/BAJ #1/6/SERI.1B//KAUZ/HEVO/3/AMAD*2/4/KIRITATI |
| 174 | 7642974 | LTP | 68.111/RGB-U//WARD RESEL/3/STIL/4/AE.SQUARROSA (188)/5/WBLL1/KUKUNA//TACUPETO F2001/3/BAJ #1/6/SERI.1B//KAUZ/HEVO/3/AMAD*2/4/KIRITATI |
| 175 | 7642977 | LTP | 68.111/RGB-U//WARD RESEL/3/STIL/4/AE.SQUARROSA (188)/5/WBLL1/KUKUNA//TACUPETO F2001/3/BAJ #1/6/SERI.1B//KAUZ/HEVO/3/AMAD*2/4/KIRITATI |
| 176 | 7642981 | LTP | 68.111/RGB-U//WARD RESEL/3/STIL/4/AE.SQUARROSA (188)/5/WBLL1/KUKUNA//TACUPETO F2001/3/BAJ #1/6/SERI.1B//KAUZ/HEVO/3/AMAD*2/4/KIRITATI |
| 177 | 7642982 | LTP | 68.111/RGB-U//WARD RESEL/3/STIL/4/AE.SQUARROSA (188)/5/WBLL1/KUKUNA//TACUPETO F2001/3/BAJ #1/6/SERI.1B//KAUZ/HEVO/3/AMAD*2/4/KIRITATI |
| 178 | 7643031 | LTP | PI 227948/4/WBLL1/KUKUNA//TACUPETO F2001/3/BAJ #1/5/SERI.1B//KAUZ/HEVO/3/AMAD*2/4/KIRITATI |
| 179 | 7643046 | LTP | IWA8611400/4/WBLL1/KUKUNA//TACUPETO F2001/3/BAJ #1/5/SERI.1B//KAUZ/HEVO/3/AMAD*2/4/KIRITATI |
| 180 | 7643055 | LTP | IWA8611400/4/WBLL1/KUKUNA//TACUPETO F2001/3/BAJ #1/5/SERI.1B//KAUZ/HEVO/3/AMAD*2/4/KIRITATI |
| 181 | 7643057 | LTP | IWA8611400/4/WBLL1/KUKUNA//TACUPETO F2001/3/BAJ #1/5/SERI.1B//KAUZ/HEVO/3/AMAD*2/4/KIRITATI |
| 182 | 7643058 | LTP | IWA8611400/4/WBLL1/KUKUNA//TACUPETO F2001/3/BAJ #1/5/SERI.1B//KAUZ/HEVO/3/AMAD*2/4/KIRITATI |
| 183 | 7643063 | LTP | IWA8611400/4/WBLL1/KUKUNA//TACUPETO F2001/3/BAJ #1/5/SERI.1B//KAUZ/HEVO/3/AMAD*2/4/KIRITATI |
| 184 | 7643074 | LTP | CHEN/AE.SQ//2*OPATA/5/SERI.1B//KAUZ/HEVO/3/AMAD*2/4/KIRITATI/6/FRET2*2/4/SNI/TRAP#1/3/KAUZ*2/TRAP//KAUZ/5/KACHU |
| 185 | 7643076 | LTP | CHEN/AE.SQ//2*OPATA/5/SERI.1B//KAUZ/HEVO/3/AMAD*2/4/KIRITATI/6/FRET2*2/4/SNI/TRAP#1/3/KAUZ*2/TRAP//KAUZ/5/KACHU |
| 186 | 7643080 | LTP | CHEN/AE.SQ//2*OPATA/5/SERI.1B//KAUZ/HEVO/3/AMAD*2/4/KIRITATI/6/FRET2*2/4/SNI/TRAP#1/3/KAUZ*2/TRAP//KAUZ/5/KACHU |
| 187 | 7643081 | LTP | CHEN/AE.SQ//2*OPATA/5/SERI.1B//KAUZ/HEVO/3/AMAD*2/4/KIRITATI/6/FRET2*2/4/SNI/TRAP#1/3/KAUZ*2/TRAP//KAUZ/5/KACHU |
| 188 | 7643083 | LTP | CHEN/AE.SQ//2*OPATA/5/SERI.1B//KAUZ/HEVO/3/AMAD*2/4/KIRITATI/6/FRET2*2/4/SNI/TRAP#1/3/KAUZ*2/TRAP//KAUZ/5/KACHU |
| 189 | 7643084 | LTP | CHEN/AE.SQ//2*OPATA/5/SERI.1B//KAUZ/HEVO/3/AMAD*2/4/KIRITATI/6/FRET2*2/4/SNI/TRAP#1/3/KAUZ*2/TRAP//KAUZ/5/KACHU |
| 190 | 7643088 | LTP | CHEN/AE.SQ//2*OPATA/5/SERI.1B//KAUZ/HEVO/3/AMAD*2/4/KIRITATI/6/FRET2*2/4/SNI/TRAP#1/3/KAUZ*2/TRAP//KAUZ/5/KACHU |
| 191 | 7643090 | LTP | CHEN/AE.SQ//2*OPATA/5/SERI.1B//KAUZ/HEVO/3/AMAD*2/4/KIRITATI/6/FRET2*2/4/SNI/TRAP#1/3/KAUZ*2/TRAP//KAUZ/5/KACHU |
| 192 | 7643091 | LTP | CHEN/AE.SQ//2*OPATA/5/SERI.1B//KAUZ/HEVO/3/AMAD*2/4/KIRITATI/6/FRET2*2/4/SNI/TRAP#1/3/KAUZ*2/TRAP//KAUZ/5/KACHU |
| 193 | 7643092 | LTP | CHEN/AE.SQ//2*OPATA/5/SERI.1B//KAUZ/HEVO/3/AMAD*2/4/KIRITATI/6/FRET2*2/4/SNI/TRAP#1/3/KAUZ*2/TRAP//KAUZ/5/KACHU |
| 194 | 7643093 | LTP | CHEN/AE.SQ//2*OPATA/5/SERI.1B//KAUZ/HEVO/3/AMAD*2/4/KIRITATI/6/FRET2*2/4/SNI/TRAP#1/3/KAUZ*2/TRAP//KAUZ/5/KACHU |
| 195 | 7643097 | LTP | CHEN/AE.SQ//2*OPATA/5/SERI.1B//KAUZ/HEVO/3/AMAD*2/4/KIRITATI/6/FRET2*2/4/SNI/TRAP#1/3/KAUZ*2/TRAP//KAUZ/5/KACHU |
| 196 | 7643099 | LTP | CHEN/AE.SQ//2*OPATA/5/SERI.1B//KAUZ/HEVO/3/AMAD*2/4/KIRITATI/6/FRET2*2/4/SNI/TRAP#1/3/KAUZ*2/TRAP//KAUZ/5/KACHU |
| 197 | 7643100 | LTP | CHEN/AE.SQ//2*OPATA/5/SERI.1B//KAUZ/HEVO/3/AMAD*2/4/KIRITATI/6/FRET2*2/4/SNI/TRAP#1/3/KAUZ*2/TRAP//KAUZ/5/KACHU |
| 198 | 7643103 | LTP | CHEN/AE.SQ//2*OPATA/5/SERI.1B//KAUZ/HEVO/3/AMAD*2/4/KIRITATI/6/FRET2*2/4/SNI/TRAP#1/3/KAUZ*2/TRAP//KAUZ/5/KACHU |
| 199 | 7643104 | LTP | CHEN/AE.SQ//2*OPATA/5/SERI.1B//KAUZ/HEVO/3/AMAD*2/4/KIRITATI/6/FRET2*2/4/SNI/TRAP#1/3/KAUZ*2/TRAP//KAUZ/5/KACHU |
| 200 | 7643108 | LTP | CHEN/AE.SQ//2*OPATA/5/SERI.1B//KAUZ/HEVO/3/AMAD*2/4/KIRITATI/6/FRET2*2/4/SNI/TRAP#1/3/KAUZ*2/TRAP//KAUZ/5/KACHU |
| 201 | 7643114 | LTP | CHEN/AE.SQ//2*OPATA/5/SERI.1B//KAUZ/HEVO/3/AMAD*2/4/KIRITATI/6/FRET2*2/4/SNI/TRAP#1/3/KAUZ*2/TRAP//KAUZ/5/KACHU |
| 202 | 7643116 | LTP | CHEN/AE.SQ//WEAVER/5/SERI.1B//KAUZ/HEVO/3/AMAD*2/4/KIRITATI/6/FRET2*2/4/SNI/TRAP#1/3/KAUZ*2/TRAP//KAUZ/5/KACHU |
| 203 | 7643119 | LTP | CHEN/AE.SQ//WEAVER/5/SERI.1B//KAUZ/HEVO/3/AMAD*2/4/KIRITATI/6/FRET2*2/4/SNI/TRAP#1/3/KAUZ*2/TRAP//KAUZ/5/KACHU |
| 204 | 7643120 | LTP | CHEN/AE.SQ//WEAVER/5/SERI.1B//KAUZ/HEVO/3/AMAD*2/4/KIRITATI/6/FRET2*2/4/SNI/TRAP#1/3/KAUZ*2/TRAP//KAUZ/5/KACHU |
| 205 | 7643125 | LTP | CHEN/AE.SQ//WEAVER/5/SERI.1B//KAUZ/HEVO/3/AMAD*2/4/KIRITATI/6/FRET2*2/4/SNI/TRAP#1/3/KAUZ*2/TRAP//KAUZ/5/KACHU |
| 206 | 7643126 | LTP | CHEN/AE.SQ//WEAVER/5/SERI.1B//KAUZ/HEVO/3/AMAD*2/4/KIRITATI/6/FRET2*2/4/SNI/TRAP#1/3/KAUZ*2/TRAP//KAUZ/5/KACHU |
| 207 | 7643133 | LTP | CHEN/AE.SQ//WEAVER/5/SERI.1B//KAUZ/HEVO/3/AMAD*2/4/KIRITATI/6/FRET2*2/4/SNI/TRAP#1/3/KAUZ*2/TRAP//KAUZ/5/KACHU |
| 208 | 7643134 | LTP | CHEN/AE.SQ//WEAVER/5/SERI.1B//KAUZ/HEVO/3/AMAD*2/4/KIRITATI/6/FRET2*2/4/SNI/TRAP#1/3/KAUZ*2/TRAP//KAUZ/5/KACHU |
| 209 | 7643135 | LTP | CHEN/AE.SQ//WEAVER/5/SERI.1B//KAUZ/HEVO/3/AMAD*2/4/KIRITATI/6/FRET2*2/4/SNI/TRAP#1/3/KAUZ*2/TRAP//KAUZ/5/KACHU |
| 210 | 7643139 | LTP | CHEN/AE.SQ//WEAVER/5/SERI.1B//KAUZ/HEVO/3/AMAD*2/4/KIRITATI/6/FRET2*2/4/SNI/TRAP#1/3/KAUZ*2/TRAP//KAUZ/5/KACHU |
| 211 | 7643143 | LTP | CHEN/AE.SQ//WEAVER/5/SERI.1B//KAUZ/HEVO/3/AMAD*2/4/KIRITATI/6/FRET2*2/4/SNI/TRAP#1/3/KAUZ*2/TRAP//KAUZ/5/KACHU |
| 212 | 7643144 | LTP | CHEN/AE.SQ//WEAVER/5/SERI.1B//KAUZ/HEVO/3/AMAD*2/4/KIRITATI/6/FRET2*2/4/SNI/TRAP#1/3/KAUZ*2/TRAP//KAUZ/5/KACHU |
| 213 | 7643145 | LTP | CHEN/AE.SQ//WEAVER/5/SERI.1B//KAUZ/HEVO/3/AMAD*2/4/KIRITATI/6/FRET2*2/4/SNI/TRAP#1/3/KAUZ*2/TRAP//KAUZ/5/KACHU |
| 214 | 7643146 | LTP | CHEN/AE.SQ//WEAVER/5/SERI.1B//KAUZ/HEVO/3/AMAD*2/4/KIRITATI/6/FRET2*2/4/SNI/TRAP#1/3/KAUZ*2/TRAP//KAUZ/5/KACHU |
| 215 | 7643150 | LTP | CHEN/AE.SQ//WEAVER/5/SERI.1B//KAUZ/HEVO/3/AMAD*2/4/KIRITATI/6/FRET2*2/4/SNI/TRAP#1/3/KAUZ*2/TRAP//KAUZ/5/KACHU |
| 216 | 7643152 | LTP | CHEN/AE.SQ//WEAVER/5/SERI.1B//KAUZ/HEVO/3/AMAD*2/4/KIRITATI/6/FRET2*2/4/SNI/TRAP#1/3/KAUZ*2/TRAP//KAUZ/5/KACHU |
| 217 | 7643155 | LTP | CHEN/AE.SQ//WEAVER/5/SERI.1B//KAUZ/HEVO/3/AMAD*2/4/KIRITATI/6/FRET2*2/4/SNI/TRAP#1/3/KAUZ*2/TRAP//KAUZ/5/KACHU |
| 218 | 7643156 | LTP | CHEN/AE.SQ//WEAVER/5/SERI.1B//KAUZ/HEVO/3/AMAD*2/4/KIRITATI/6/FRET2*2/4/SNI/TRAP#1/3/KAUZ*2/TRAP//KAUZ/5/KACHU |
| 219 | 7643157 | LTP | CHEN/AE.SQ//WEAVER/5/SERI.1B//KAUZ/HEVO/3/AMAD*2/4/KIRITATI/6/FRET2*2/4/SNI/TRAP#1/3/KAUZ*2/TRAP//KAUZ/5/KACHU |
| 220 | 7643160 | LTP | CHEN/AE.SQ//WEAVER/5/SERI.1B//KAUZ/HEVO/3/AMAD*2/4/KIRITATI/6/FRET2*2/4/SNI/TRAP#1/3/KAUZ*2/TRAP//KAUZ/5/KACHU |
| 221 | 7643162 | LTP | CHEN/AE.SQ//WEAVER/5/SERI.1B//KAUZ/HEVO/3/AMAD*2/4/KIRITATI/6/FRET2*2/4/SNI/TRAP#1/3/KAUZ*2/TRAP//KAUZ/5/KACHU |
| 222 | 7643168 | LTP | SABUF/4/ALTAR 84/AE.SQUARROSA (224)//CUPE/3/CROC_1/AE.SQUARROSA (205)//F27202/5/SERI.1B//KAUZ/HEVO/3/AMAD*2/4/KIRITATI/6/FRET2*2/4/SNI/TRAP#1/3/KAUZ*2/TRAP//KAUZ/5/KACHU |
| 223 | 7643169 | LTP | SABUF/4/ALTAR 84/AE.SQUARROSA (224)//CUPE/3/CROC_1/AE.SQUARROSA (205)//F27202/5/SERI.1B//KAUZ/HEVO/3/AMAD*2/4/KIRITATI/6/FRET2*2/4/SNI/TRAP#1/3/KAUZ*2/TRAP//KAUZ/5/KACHU |
| 224 | 7643174 | LTP | SABUF/4/ALTAR 84/AE.SQUARROSA (224)//CUPE/3/CROC_1/AE.SQUARROSA (205)//F27202/5/SERI.1B//KAUZ/HEVO/3/AMAD*2/4/KIRITATI/6/FRET2*2/4/SNI/TRAP#1/3/KAUZ*2/TRAP//KAUZ/5/KACHU |
| 225 | 7643177 | LTP | SABUF/4/ALTAR 84/AE.SQUARROSA (224)//CUPE/3/CROC_1/AE.SQUARROSA (205)//F27202/5/SERI.1B//KAUZ/HEVO/3/AMAD*2/4/KIRITATI/6/FRET2*2/4/SNI/TRAP#1/3/KAUZ*2/TRAP//KAUZ/5/KACHU |
| 226 | 7643188 | LTP | SABUF/4/ALTAR 84/AE.SQUARROSA (224)//CUPE/3/CROC_1/AE.SQUARROSA (205)//F27202/5/SERI.1B//KAUZ/HEVO/3/AMAD*2/4/KIRITATI/6/FRET2*2/4/SNI/TRAP#1/3/KAUZ*2/TRAP//KAUZ/5/KACHU |
| 227 | 7643189 | LTP | SABUF/4/ALTAR 84/AE.SQUARROSA (224)//CUPE/3/CROC_1/AE.SQUARROSA (205)//F27202/5/SERI.1B//KAUZ/HEVO/3/AMAD*2/4/KIRITATI/6/FRET2*2/4/SNI/TRAP#1/3/KAUZ*2/TRAP//KAUZ/5/KACHU |
| 228 | 7643198 | LTP | SABUF/4/ALTAR 84/AE.SQUARROSA (224)//CUPE/3/CROC_1/AE.SQUARROSA (205)//F27202/5/SERI.1B//KAUZ/HEVO/3/AMAD*2/4/KIRITATI/6/FRET2*2/4/SNI/TRAP#1/3/KAUZ*2/TRAP//KAUZ/5/KACHU |
| 229 | 7643201 | LTP | CHEN/AE.SQ//2*OPATA/5/SERI.1B//KAUZ/HEVO/3/AMAD*2/4/KIRITATI/6/FRET2*2/4/SNI/TRAP#1/3/KAUZ*2/TRAP//KAUZ/5/KACHU |
| 230 | 7643203 | LTP | CHEN/AE.SQ//2*OPATA/5/SERI.1B//KAUZ/HEVO/3/AMAD*2/4/KIRITATI/6/FRET2*2/4/SNI/TRAP#1/3/KAUZ*2/TRAP//KAUZ/5/KACHU |
| 231 | 7643204 | LTP | CHEN/AE.SQ//2*OPATA/5/SERI.1B//KAUZ/HEVO/3/AMAD*2/4/KIRITATI/6/FRET2*2/4/SNI/TRAP#1/3/KAUZ*2/TRAP//KAUZ/5/KACHU |
| 232 | 7643206 | LTP | CHEN/AE.SQ//2*OPATA/5/SERI.1B//KAUZ/HEVO/3/AMAD*2/4/KIRITATI/6/FRET2*2/4/SNI/TRAP#1/3/KAUZ*2/TRAP//KAUZ/5/KACHU |
| 233 | 7643207 | LTP | CHEN/AE.SQ//2*OPATA/5/SERI.1B//KAUZ/HEVO/3/AMAD*2/4/KIRITATI/6/FRET2*2/4/SNI/TRAP#1/3/KAUZ*2/TRAP//KAUZ/5/KACHU |
| 234 | 7643228 | LTP | CHEN/AE.SQ//2*OPATA/5/SERI.1B//KAUZ/HEVO/3/AMAD*2/4/KIRITATI/6/FRET2*2/4/SNI/TRAP#1/3/KAUZ*2/TRAP//KAUZ/5/KACHU |
| 235 | 7643229 | LTP | CHEN/AE.SQ//2*OPATA/5/SERI.1B//KAUZ/HEVO/3/AMAD*2/4/KIRITATI/6/FRET2*2/4/SNI/TRAP#1/3/KAUZ*2/TRAP//KAUZ/5/KACHU |
| 236 | 7643238 | LTP | CHEN/AE.SQ//2*OPATA/5/SERI.1B//KAUZ/HEVO/3/AMAD*2/4/KIRITATI/6/FRET2*2/4/SNI/TRAP#1/3/KAUZ*2/TRAP//KAUZ/5/KACHU |
| 237 | 7643253 | LTP | CHEN/AE.SQ//2*OPATA/5/SERI.1B//KAUZ/HEVO/3/AMAD*2/4/KIRITATI/6/FRET2*2/4/SNI/TRAP#1/3/KAUZ*2/TRAP//KAUZ/5/KACHU |
| 238 | 7643257 | LTP | CHEN/AE.SQ//2*OPATA/5/SERI.1B//KAUZ/HEVO/3/AMAD*2/4/KIRITATI/6/FRET2*2/4/SNI/TRAP#1/3/KAUZ*2/TRAP//KAUZ/5/KACHU |
| 239 | 7643263 | LTP | CHEN/AE.SQ//2*OPATA/5/SERI.1B//KAUZ/HEVO/3/AMAD*2/4/KIRITATI/6/FRET2*2/4/SNI/TRAP#1/3/KAUZ*2/TRAP//KAUZ/5/KACHU |
| 240 | 7643272 | LTP | SABUF/4/ALTAR 84/AE.SQUARROSA (224)//CUPE/3/CROC_1/AE.SQUARROSA (205)//F27202/5/SERI.1B//KAUZ/HEVO/3/AMAD*2/4/KIRITATI/6/FRET2*2/4/SNI/TRAP#1/3/KAUZ*2/TRAP//KAUZ/5/KACHU |
| 241 | 7643274 | LTP | SABUF/4/ALTAR 84/AE.SQUARROSA (224)//CUPE/3/CROC_1/AE.SQUARROSA (205)//F27202/5/SERI.1B//KAUZ/HEVO/3/AMAD*2/4/KIRITATI/6/FRET2*2/4/SNI/TRAP#1/3/KAUZ*2/TRAP//KAUZ/5/KACHU |
| 242 | 7643276 | LTP | SABUF/4/ALTAR 84/AE.SQUARROSA (224)//CUPE/3/CROC_1/AE.SQUARROSA (205)//F27202/5/SERI.1B//KAUZ/HEVO/3/AMAD*2/4/KIRITATI/6/FRET2*2/4/SNI/TRAP#1/3/KAUZ*2/TRAP//KAUZ/5/KACHU |
| 243 | 7643279 | LTP | SABUF/4/ALTAR 84/AE.SQUARROSA (224)//CUPE/3/CROC_1/AE.SQUARROSA (205)//F27202/5/SERI.1B//KAUZ/HEVO/3/AMAD*2/4/KIRITATI/6/FRET2*2/4/SNI/TRAP#1/3/KAUZ*2/TRAP//KAUZ/5/KACHU |
| 244 | 7643280 | LTP | SABUF/4/ALTAR 84/AE.SQUARROSA (224)//CUPE/3/CROC_1/AE.SQUARROSA (205)//F27202/5/SERI.1B//KAUZ/HEVO/3/AMAD*2/4/KIRITATI/6/FRET2*2/4/SNI/TRAP#1/3/KAUZ*2/TRAP//KAUZ/5/KACHU |
| 245 | 7643287 | LTP | SABUF/4/ALTAR 84/AE.SQUARROSA (224)//CUPE/3/CROC_1/AE.SQUARROSA (205)//F27202/5/SERI.1B//KAUZ/HEVO/3/AMAD*2/4/KIRITATI/6/FRET2*2/4/SNI/TRAP#1/3/KAUZ*2/TRAP//KAUZ/5/KACHU |
| 246 | 7643299 | LTP | IG 41506/5/SERI.1B//KAUZ/HEVO/3/AMAD*2/4/KIRITATI/6/FRET2*2/4/SNI/TRAP#1/3/KAUZ*2/TRAP//KAUZ/5/KACHU |
| 247 | 7643306 | LTP | IG 41506/5/SERI.1B//KAUZ/HEVO/3/AMAD*2/4/KIRITATI/6/FRET2*2/4/SNI/TRAP#1/3/KAUZ*2/TRAP//KAUZ/5/KACHU |
| 248 | 7643309 | LTP | IG 41506/5/SERI.1B//KAUZ/HEVO/3/AMAD*2/4/KIRITATI/6/FRET2*2/4/SNI/TRAP#1/3/KAUZ*2/TRAP//KAUZ/5/KACHU |
| 249 | 7643311 | LTP | IG 41506/5/SERI.1B//KAUZ/HEVO/3/AMAD*2/4/KIRITATI/6/FRET2*2/4/SNI/TRAP#1/3/KAUZ*2/TRAP//KAUZ/5/KACHU |
| 250 | 7643316 | LTP | IG 41506/5/SERI.1B//KAUZ/HEVO/3/AMAD*2/4/KIRITATI/6/FRET2*2/4/SNI/TRAP#1/3/KAUZ*2/TRAP//KAUZ/5/KACHU |
| 251 | 7643317 | LTP | IG 41506/5/SERI.1B//KAUZ/HEVO/3/AMAD*2/4/KIRITATI/6/FRET2*2/4/SNI/TRAP#1/3/KAUZ*2/TRAP//KAUZ/5/KACHU |
| 252 | 7643333 | LTP | IG 41514/5/SERI.1B//KAUZ/HEVO/3/AMAD*2/4/KIRITATI/6/FRET2*2/4/SNI/TRAP#1/3/KAUZ*2/TRAP//KAUZ/5/KACHU |
| 253 | 7643343 | LTP | IG 41514/5/SERI.1B//KAUZ/HEVO/3/AMAD*2/4/KIRITATI/6/FRET2*2/4/SNI/TRAP#1/3/KAUZ*2/TRAP//KAUZ/5/KACHU |
| 254 | 7643346 | LTP | IG 41514/5/SERI.1B//KAUZ/HEVO/3/AMAD*2/4/KIRITATI/6/FRET2*2/4/SNI/TRAP#1/3/KAUZ*2/TRAP//KAUZ/5/KACHU |
| 255 | 7643348 | LTP | IG 41514/5/SERI.1B//KAUZ/HEVO/3/AMAD*2/4/KIRITATI/6/FRET2*2/4/SNI/TRAP#1/3/KAUZ*2/TRAP//KAUZ/5/KACHU |
| 256 | 7643349 | LTP | IG 41514/5/SERI.1B//KAUZ/HEVO/3/AMAD*2/4/KIRITATI/6/FRET2*2/4/SNI/TRAP#1/3/KAUZ*2/TRAP//KAUZ/5/KACHU |
| 257 | 7643355 | LTP | IG 41514/5/SERI.1B//KAUZ/HEVO/3/AMAD*2/4/KIRITATI/6/FRET2*2/4/SNI/TRAP#1/3/KAUZ*2/TRAP//KAUZ/5/KACHU |
| 258 | 7643357 | LTP | IG 41514/5/SERI.1B//KAUZ/HEVO/3/AMAD*2/4/KIRITATI/6/FRET2*2/4/SNI/TRAP#1/3/KAUZ*2/TRAP//KAUZ/5/KACHU |
| 259 | 7643358 | LTP | IG 41514/5/SERI.1B//KAUZ/HEVO/3/AMAD*2/4/KIRITATI/6/FRET2*2/4/SNI/TRAP#1/3/KAUZ*2/TRAP//KAUZ/5/KACHU |
| 260 | 7643362 | LTP | IG 41514/5/SERI.1B//KAUZ/HEVO/3/AMAD*2/4/KIRITATI/6/FRET2*2/4/SNI/TRAP#1/3/KAUZ*2/TRAP//KAUZ/5/KACHU |
| 261 | 7643363 | LTP | IG 41514/5/SERI.1B//KAUZ/HEVO/3/AMAD*2/4/KIRITATI/6/FRET2*2/4/SNI/TRAP#1/3/KAUZ*2/TRAP//KAUZ/5/KACHU |
| 262 | 7643367 | LTP | IG 41514/5/SERI.1B//KAUZ/HEVO/3/AMAD*2/4/KIRITATI/6/FRET2*2/4/SNI/TRAP#1/3/KAUZ*2/TRAP//KAUZ/5/KACHU |
| 263 | 7643368 | LTP | IG 41514/5/SERI.1B//KAUZ/HEVO/3/AMAD*2/4/KIRITATI/6/FRET2*2/4/SNI/TRAP#1/3/KAUZ*2/TRAP//KAUZ/5/KACHU |
| 264 | 7643370 | LTP | IG 41514/5/SERI.1B//KAUZ/HEVO/3/AMAD*2/4/KIRITATI/6/FRET2*2/4/SNI/TRAP#1/3/KAUZ*2/TRAP//KAUZ/5/KACHU |
| 265 | 7643383 | LTP | IG 41654/5/SERI.1B//KAUZ/HEVO/3/AMAD*2/4/KIRITATI/6/FRET2*2/4/SNI/TRAP#1/3/KAUZ*2/TRAP//KAUZ/5/KACHU |
| 266 | 7643385 | LTP | IG 41654/5/SERI.1B//KAUZ/HEVO/3/AMAD*2/4/KIRITATI/6/FRET2*2/4/SNI/TRAP#1/3/KAUZ*2/TRAP//KAUZ/5/KACHU |
| 267 | 7643390 | LTP | IG 41654/5/SERI.1B//KAUZ/HEVO/3/AMAD*2/4/KIRITATI/6/FRET2*2/4/SNI/TRAP#1/3/KAUZ*2/TRAP//KAUZ/5/KACHU |
| 268 | 7643392 | LTP | IG 41654/5/SERI.1B//KAUZ/HEVO/3/AMAD*2/4/KIRITATI/6/FRET2*2/4/SNI/TRAP#1/3/KAUZ*2/TRAP//KAUZ/5/KACHU |
| 269 | 7643395 | LTP | IG 41654/5/SERI.1B//KAUZ/HEVO/3/AMAD*2/4/KIRITATI/6/FRET2*2/4/SNI/TRAP#1/3/KAUZ*2/TRAP//KAUZ/5/KACHU |
| 270 | 7643400 | LTP | IG 41654/5/SERI.1B//KAUZ/HEVO/3/AMAD*2/4/KIRITATI/6/FRET2*2/4/SNI/TRAP#1/3/KAUZ*2/TRAP//KAUZ/5/KACHU |
| 271 | 7643401 | LTP | IG 41654/5/SERI.1B//KAUZ/HEVO/3/AMAD*2/4/KIRITATI/6/FRET2*2/4/SNI/TRAP#1/3/KAUZ*2/TRAP//KAUZ/5/KACHU |
| 272 | 7643411 | LTP | IG 131673/5/SERI.1B//KAUZ/HEVO/3/AMAD*2/4/KIRITATI/6/FRET2*2/4/SNI/TRAP#1/3/KAUZ*2/TRAP//KAUZ/5/KACHU |
| 273 | 7643413 | LTP | IG 131673/5/SERI.1B//KAUZ/HEVO/3/AMAD*2/4/KIRITATI/6/FRET2*2/4/SNI/TRAP#1/3/KAUZ*2/TRAP//KAUZ/5/KACHU |
| 274 | 7643416 | LTP | IG 131673/5/SERI.1B//KAUZ/HEVO/3/AMAD*2/4/KIRITATI/6/FRET2*2/4/SNI/TRAP#1/3/KAUZ*2/TRAP//KAUZ/5/KACHU |
| 275 | 7643419 | LTP | IG 131673/5/SERI.1B//KAUZ/HEVO/3/AMAD*2/4/KIRITATI/6/FRET2*2/4/SNI/TRAP#1/3/KAUZ*2/TRAP//KAUZ/5/KACHU |
| 276 | 7643420 | LTP | IG 131673/5/SERI.1B//KAUZ/HEVO/3/AMAD*2/4/KIRITATI/6/FRET2*2/4/SNI/TRAP#1/3/KAUZ*2/TRAP//KAUZ/5/KACHU |
| 277 | 7643422 | LTP | IG 131673/5/SERI.1B//KAUZ/HEVO/3/AMAD*2/4/KIRITATI/6/FRET2*2/4/SNI/TRAP#1/3/KAUZ*2/TRAP//KAUZ/5/KACHU |
| 278 | 7643423 | LTP | IG 131673/5/SERI.1B//KAUZ/HEVO/3/AMAD*2/4/KIRITATI/6/FRET2*2/4/SNI/TRAP#1/3/KAUZ*2/TRAP//KAUZ/5/KACHU |
| 279 | 7643425 | LTP | IG 131673/5/SERI.1B//KAUZ/HEVO/3/AMAD*2/4/KIRITATI/6/FRET2*2/4/SNI/TRAP#1/3/KAUZ*2/TRAP//KAUZ/5/KACHU |
| 280 | 7643430 | LTP | IG 131673/5/SERI.1B//KAUZ/HEVO/3/AMAD*2/4/KIRITATI/6/FRET2*2/4/SNI/TRAP#1/3/KAUZ*2/TRAP//KAUZ/5/KACHU |
| 281 | 7643433 | LTP | IG 131673/5/SERI.1B//KAUZ/HEVO/3/AMAD*2/4/KIRITATI/6/FRET2*2/4/SNI/TRAP#1/3/KAUZ*2/TRAP//KAUZ/5/KACHU |
| 282 | 7643442 | LTP | IG 131673/5/SERI.1B//KAUZ/HEVO/3/AMAD*2/4/KIRITATI/6/FRET2*2/4/SNI/TRAP#1/3/KAUZ*2/TRAP//KAUZ/5/KACHU |
| 283 | 7643446 | LTP | IG 131673/5/SERI.1B//KAUZ/HEVO/3/AMAD*2/4/KIRITATI/6/FRET2*2/4/SNI/TRAP#1/3/KAUZ*2/TRAP//KAUZ/5/KACHU |
| 284 | 7643449 | LTP | IG 131673/5/SERI.1B//KAUZ/HEVO/3/AMAD*2/4/KIRITATI/6/FRET2*2/4/SNI/TRAP#1/3/KAUZ*2/TRAP//KAUZ/5/KACHU |
| 285 | 7643458 | LTP | IG 131673/5/SERI.1B//KAUZ/HEVO/3/AMAD*2/4/KIRITATI/6/FRET2*2/4/SNI/TRAP#1/3/KAUZ*2/TRAP//KAUZ/5/KACHU |
| 286 | 7643466 | LTP | INDIA-58/5/SERI.1B//KAUZ/HEVO/3/AMAD*2/4/KIRITATI/6/FRET2*2/4/SNI/TRAP#1/3/KAUZ*2/TRAP//KAUZ/5/KACHU |
| 287 | 7643478 | LTP | INDIA-58/5/SERI.1B//KAUZ/HEVO/3/AMAD*2/4/KIRITATI/6/FRET2*2/4/SNI/TRAP#1/3/KAUZ*2/TRAP//KAUZ/5/KACHU |
| 288 | 7643496 | LTP | IG 41783/5/SERI.1B//KAUZ/HEVO/3/AMAD*2/4/KIRITATI/6/FRET2*2/4/SNI/TRAP#1/3/KAUZ*2/TRAP//KAUZ/5/KACHU |
| 289 | 7643512 | LTP | IG 42132/5/SERI.1B//KAUZ/HEVO/3/AMAD*2/4/KIRITATI/6/FRET2*2/4/SNI/TRAP#1/3/KAUZ*2/TRAP//KAUZ/5/KACHU |
| 290 | 7643521 | LTP | INDIA-209/5/SERI.1B//KAUZ/HEVO/3/AMAD*2/4/KIRITATI/6/FRET2*2/4/SNI/TRAP#1/3/KAUZ*2/TRAP//KAUZ/5/KACHU |
| 291 | 7643526 | LTP | INDIA-209/5/SERI.1B//KAUZ/HEVO/3/AMAD*2/4/KIRITATI/6/FRET2*2/4/SNI/TRAP#1/3/KAUZ*2/TRAP//KAUZ/5/KACHU |
| 292 | 7643531 | LTP | INDIA-209/5/SERI.1B//KAUZ/HEVO/3/AMAD*2/4/KIRITATI/6/FRET2*2/4/SNI/TRAP#1/3/KAUZ*2/TRAP//KAUZ/5/KACHU |
| 293 | 7643533 | LTP | INDIA-209/5/SERI.1B//KAUZ/HEVO/3/AMAD*2/4/KIRITATI/6/FRET2*2/4/SNI/TRAP#1/3/KAUZ*2/TRAP//KAUZ/5/KACHU |
| 294 | 7643537 | LTP | IRAQ-21/5/SERI.1B//KAUZ/HEVO/3/AMAD*2/4/KIRITATI/6/FRET2*2/4/SNI/TRAP#1/3/KAUZ*2/TRAP//KAUZ/5/KACHU |
| 295 | 7643544 | LTP | IRAQ-21/5/SERI.1B//KAUZ/HEVO/3/AMAD*2/4/KIRITATI/6/FRET2*2/4/SNI/TRAP#1/3/KAUZ*2/TRAP//KAUZ/5/KACHU |
| 296 | 7643548 | LTP | IRAQ-21/5/SERI.1B//KAUZ/HEVO/3/AMAD*2/4/KIRITATI/6/FRET2*2/4/SNI/TRAP#1/3/KAUZ*2/TRAP//KAUZ/5/KACHU |
| 297 | 7643559 | LTP | IRAQ-34/5/SERI.1B//KAUZ/HEVO/3/AMAD*2/4/KIRITATI/6/FRET2*2/4/SNI/TRAP#1/3/KAUZ*2/TRAP//KAUZ/5/KACHU |
| 298 | 7643567 | LTP | IRAQ-34/5/SERI.1B//KAUZ/HEVO/3/AMAD*2/4/KIRITATI/6/FRET2*2/4/SNI/TRAP#1/3/KAUZ*2/TRAP//KAUZ/5/KACHU |
| 299 | 7643569 | LTP | IRAQ-34/5/SERI.1B//KAUZ/HEVO/3/AMAD*2/4/KIRITATI/6/FRET2*2/4/SNI/TRAP#1/3/KAUZ*2/TRAP//KAUZ/5/KACHU |
| 300 | 7643571 | LTP | IRAQ-34/5/SERI.1B//KAUZ/HEVO/3/AMAD*2/4/KIRITATI/6/FRET2*2/4/SNI/TRAP#1/3/KAUZ*2/TRAP//KAUZ/5/KACHU |
| 301 | 7643577 | LTP | H-1496/5/SERI.1B//KAUZ/HEVO/3/AMAD*2/4/KIRITATI/6/FRET2*2/4/SNI/TRAP#1/3/KAUZ*2/TRAP//KAUZ/5/KACHU |
| 302 | 7643582 | LTP | H-1496/5/SERI.1B//KAUZ/HEVO/3/AMAD*2/4/KIRITATI/6/FRET2*2/4/SNI/TRAP#1/3/KAUZ*2/TRAP//KAUZ/5/KACHU |
| 303 | 7643587 | LTP | INDIA-227/5/SERI.1B//KAUZ/HEVO/3/AMAD*2/4/KIRITATI/6/FRET2*2/4/SNI/TRAP#1/3/KAUZ*2/TRAP//KAUZ/5/KACHU |
| 304 | 7643588 | LTP | INDIA-227/5/SERI.1B//KAUZ/HEVO/3/AMAD*2/4/KIRITATI/6/FRET2*2/4/SNI/TRAP#1/3/KAUZ*2/TRAP//KAUZ/5/KACHU |
| 305 | 7643593 | LTP | INDIA-227/5/SERI.1B//KAUZ/HEVO/3/AMAD*2/4/KIRITATI/6/FRET2*2/4/SNI/TRAP#1/3/KAUZ*2/TRAP//KAUZ/5/KACHU |
| 306 | 7643600 | LTP | INDIA-227/5/SERI.1B//KAUZ/HEVO/3/AMAD*2/4/KIRITATI/6/FRET2*2/4/SNI/TRAP#1/3/KAUZ*2/TRAP//KAUZ/5/KACHU |
| 307 | 7643601 | LTP | INDIA-227/5/SERI.1B//KAUZ/HEVO/3/AMAD*2/4/KIRITATI/6/FRET2*2/4/SNI/TRAP#1/3/KAUZ*2/TRAP//KAUZ/5/KACHU |
| 308 | 7643605 | LTP | INDIA-227/5/SERI.1B//KAUZ/HEVO/3/AMAD*2/4/KIRITATI/6/FRET2*2/4/SNI/TRAP#1/3/KAUZ*2/TRAP//KAUZ/5/KACHU |
| 309 | 7643639 | LTP | INDIA-321/5/SERI.1B//KAUZ/HEVO/3/AMAD*2/4/KIRITATI/6/FRET2*2/4/SNI/TRAP#1/3/KAUZ*2/TRAP//KAUZ/5/KACHU |
| 310 | 7643669 | LTP | ARLIN_1/AE.SQUARROSA (305)/6/FRET2*2/4/SNI/TRAP#1/3/KAUZ*2/TRAP//KAUZ/5/KACHU/7/HUW234+LR34/PRINIA*2//KIRITATI |
| 311 | 7643671 | LTP | ARLIN_1/AE.SQUARROSA (305)/6/FRET2*2/4/SNI/TRAP#1/3/KAUZ*2/TRAP//KAUZ/5/KACHU/7/HUW234+LR34/PRINIA*2//KIRITATI |
| 312 | 7643713 | LTP | MAYOOR//TK SN1081/AE.SQUARROSA (222)/6/FRET2*2/4/SNI/TRAP#1/3/KAUZ*2/TRAP//KAUZ/5/KACHU/7/HUW234+LR34/PRINIA*2//KIRITATI |
| 313 | 7643715 | LTP | MAYOOR//TK SN1081/AE.SQUARROSA (222)/6/FRET2*2/4/SNI/TRAP#1/3/KAUZ*2/TRAP//KAUZ/5/KACHU/7/HUW234+LR34/PRINIA*2//KIRITATI |
| 314 | 7643718 | LTP | MAYOOR//TK SN1081/AE.SQUARROSA (222)/6/FRET2*2/4/SNI/TRAP#1/3/KAUZ*2/TRAP//KAUZ/5/KACHU/7/HUW234+LR34/PRINIA*2//KIRITATI |
| 315 | 7643719 | LTP | MAYOOR//TK SN1081/AE.SQUARROSA (222)/6/FRET2*2/4/SNI/TRAP#1/3/KAUZ*2/TRAP//KAUZ/5/KACHU/7/HUW234+LR34/PRINIA*2//KIRITATI |
| 316 | 7643738 | LTP | IG 41613/6/FRET2*2/4/SNI/TRAP#1/3/KAUZ*2/TRAP//KAUZ/5/KACHU/7/HUW234+LR34/PRINIA*2//KIRITATI |
| 317 | 7643739 | LTP | IG 41613/6/FRET2*2/4/SNI/TRAP#1/3/KAUZ*2/TRAP//KAUZ/5/KACHU/7/HUW234+LR34/PRINIA*2//KIRITATI |
| 318 | 7643740 | LTP | IG 41613/6/FRET2*2/4/SNI/TRAP#1/3/KAUZ*2/TRAP//KAUZ/5/KACHU/7/HUW234+LR34/PRINIA*2//KIRITATI |
| 319 | 7643753 | LTP | IG 42144/6/FRET2*2/4/SNI/TRAP#1/3/KAUZ*2/TRAP//KAUZ/5/KACHU/7/HUW234+LR34/PRINIA*2//KIRITATI |
| 320 | 7643759 | LTP | IG 42144/6/FRET2*2/4/SNI/TRAP#1/3/KAUZ*2/TRAP//KAUZ/5/KACHU/7/HUW234+LR34/PRINIA*2//KIRITATI |
| 321 | 7643778 | LTP | BCN/4/RABI//GS/CRA/3/AE.SQUARROSA (895)/5/HUW234+LR34/PRINIA*2//KIRITATI/6/WHEAR/KRONSTAD F2004 |
| 322 | 7643938 | LTP | GARZA/BOY//AE.SQUARROSA (520)/3/WHEAR/KRONSTAD F2004/7/SHA7/VEE#5/5/VEE#8//JUP/BJY/3/F3.71/TRM/4/2*WEAVER/6/SKAUZ/PARUS//PARUS |
| 323 | 7643951 | LTP | DOY1/AE.SQUARROSA (532)//WHEAR/KRONSTAD F2004/7/SHA7/VEE#5/5/VEE#8//JUP/BJY/3/F3.71/TRM/4/2*WEAVER/6/SKAUZ/PARUS//PARUS |
| 324 | 7643975 | LTP | ALTAR 84/AE.SQUARROSA (502)//WHEAR/KRONSTAD F2004/7/SHA7/VEE#5/5/VEE#8//JUP/BJY/3/F3.71/TRM/4/2*WEAVER/6/SKAUZ/PARUS//PARUS |
| 325 | 7643992 | LTP | T.DICOCCON CI9309/AE.SQUARROSA (1027)//WHEAR/KRONSTAD F2004/7/SHA7/VEE#5/5/VEE#8//JUP/BJY/3/F3.71/TRM/4/2*WEAVER/6/SKAUZ/PARUS//PARUS |
| 326 | 7643997 | LTP | T.DICOCCON CI9309/AE.SQUARROSA (1027)//WHEAR/KRONSTAD F2004/7/SHA7/VEE#5/5/VEE#8//JUP/BJY/3/F3.71/TRM/4/2*WEAVER/6/SKAUZ/PARUS//PARUS |
| 327 | 7644005 | LTP | T.DICOCCON PI94625/AE.SQUARROSA (502)//WHEAR/KRONSTAD F2004/7/SHA7/VEE#5/5/VEE#8//JUP/BJY/3/F3.71/TRM/4/2*WEAVER/6/SKAUZ/PARUS//PARUS |
| 328 | 7644045 | LTP | T.DICOCCON CI3686/AE.SQUARROSA (458)//WHEAR/KRONSTAD F2004/7/SHA7/VEE#5/5/VEE#8//JUP/BJY/3/F3.71/TRM/4/2*WEAVER/6/SKAUZ/PARUS//PARUS |
| 329 | 7644054 | LTP | D67.2/PARANA 66.270//AE.SQUARROSA (465)/3/WHEAR/KRONSTAD F2004/7/SHA7/VEE#5/5/VEE#8//JUP/BJY/3/F3.71/TRM/4/2*WEAVER/6/SKAUZ/PARUS//PARUS |
| 330 | 7644127 | LTP | MEX94.13.1.47//WHEAR/KRONSTAD F2004/7/SHA7/VEE#5/5/VEE#8//JUP/BJY/3/F3.71/TRM/4/2*WEAVER/6/SKAUZ/PARUS//PARUS |
| 331 | 7644145 | LTP | OAX93.5.1.1//WHEAR/KRONSTAD F2004/7/SHA7/VEE#5/5/VEE#8//JUP/BJY/3/F3.71/TRM/4/2*WEAVER/6/SKAUZ/PARUS//PARUS |
| 332 | 7644191 | LTP | 68.111/RGB-U//WARD/3/AE.SQUARROSA (388)/7/SHA7/VEE#5/5/VEE#8//JUP/BJY/3/F3.71/TRM/4/2*WEAVER/6/SKAUZ/PARUS//PARUS/8/CNDO/R143//ENTE/MEXI_2/3/AEGILOPS SQUARROSA (TAUS)/4/WEAVER/5/PICUS/6/TROST/7/TACUPETO F2001 |
| 333 | 7644194 | LTP | 68.111/RGB-U//WARD/3/AE.SQUARROSA (388)/7/SHA7/VEE#5/5/VEE#8//JUP/BJY/3/F3.71/TRM/4/2*WEAVER/6/SKAUZ/PARUS//PARUS/8/CNDO/R143//ENTE/MEXI_2/3/AEGILOPS SQUARROSA (TAUS)/4/WEAVER/5/PICUS/6/TROST/7/TACUPETO F2001 |
| 334 | 7644197 | LTP | 68.111/RGB-U//WARD/3/AE.SQUARROSA (388)/7/SHA7/VEE#5/5/VEE#8//JUP/BJY/3/F3.71/TRM/4/2*WEAVER/6/SKAUZ/PARUS//PARUS/8/CNDO/R143//ENTE/MEXI_2/3/AEGILOPS SQUARROSA (TAUS)/4/WEAVER/5/PICUS/6/TROST/7/TACUPETO F2001 |
| 335 | 7644198 | LTP | 68.111/RGB-U//WARD/3/AE.SQUARROSA (388)/7/SHA7/VEE#5/5/VEE#8//JUP/BJY/3/F3.71/TRM/4/2*WEAVER/6/SKAUZ/PARUS//PARUS/8/CNDO/R143//ENTE/MEXI_2/3/AEGILOPS SQUARROSA (TAUS)/4/WEAVER/5/PICUS/6/TROST/7/TACUPETO F2001 |
| 336 | 7644199 | LTP | 68.111/RGB-U//WARD/3/AE.SQUARROSA (388)/7/SHA7/VEE#5/5/VEE#8//JUP/BJY/3/F3.71/TRM/4/2*WEAVER/6/SKAUZ/PARUS//PARUS/8/CNDO/R143//ENTE/MEXI_2/3/AEGILOPS SQUARROSA (TAUS)/4/WEAVER/5/PICUS/6/TROST/7/TACUPETO F2001 |
| 337 | 7644239 | LTP | ACO89/AE.SQUARROSA (290)/7/SHA7/VEE#5/5/VEE#8//JUP/BJY/3/F3.71/TRM/4/2*WEAVER/6/SKAUZ/PARUS//PARUS/8/CNDO/R143//ENTE/MEXI_2/3/AEGILOPS SQUARROSA (TAUS)/4/WEAVER/5/PICUS/6/TROST/7/TACUPETO F2001 |
| 338 | 7644275 | LTP | PERSIA-101/7/SHA7/VEE#5/5/VEE#8//JUP/BJY/3/F3.71/TRM/4/2*WEAVER/6/SKAUZ/PARUS//PARUS/8/CNDO/R143//ENTE/MEXI_2/3/AEGILOPS SQUARROSA (TAUS)/4/WEAVER/5/PICUS/6/TROST/7/TACUPETO F2001 |
| 339 | 7644415 | LTP | GARZA/BOY//AE.SQUARROSA (170)/6/KAUZ//ALTAR 84/AOS/3/PASTOR/4/MILAN/CUPE//SW89.3064/5/KIRITATI/7/SW89.5277/BORL95//SKAUZ/3/PRL/2*PASTOR/4/HEILO |
| 340 | 7644417 | LTP | BCN//CETA/AE.SEARSII (34D)/6/KAUZ//ALTAR 84/AOS/3/PASTOR/4/MILAN/CUPE//SW89.3064/5/KIRITATI/7/SW89.5277/BORL95//SKAUZ/3/PRL/2*PASTOR/4/HEILO |
| 341 | 7644421 | LTP | BCN//CETA/AE.SEARSII (34D)/6/KAUZ//ALTAR 84/AOS/3/PASTOR/4/MILAN/CUPE//SW89.3064/5/KIRITATI/7/SW89.5277/BORL95//SKAUZ/3/PRL/2*PASTOR/4/HEILO |
| 342 | 7644491 | LTP | CHEN/AE.SQ//2*OPATA/6/KAUZ//ALTAR 84/AOS/3/PASTOR/4/MILAN/CUPE//SW89.3064/5/KIRITATI/7/SW89.5277/BORL95//SKAUZ/3/PRL/2*PASTOR/4/HEILO |
| 343 | 7644526 | LTP | IG 43223/6/KAUZ//ALTAR 84/AOS/3/PASTOR/4/MILAN/CUPE//SW89.3064/5/KIRITATI/7/SW89.5277/BORL95//SKAUZ/3/PRL/2*PASTOR/4/HEILO |
| 344 | 7644637 | LTP | 68.111/RGB-U//WARD/3/FGO/4/RABI/5/AE.SQUARROSA (878)/6/KAUZ//ALTAR 84/AOS/3/PASTOR/4/MILAN/CUPE//SW89.3064/5/KIRITATI/7/SW89.5277/BORL95//SKAUZ/3/PRL/2*PASTOR/4/HEILO |
| 345 | 7644714 | LTP | GARZA/BOY//AE.SQUARROSA (428)/5/SW89.5277/BORL95//SKAUZ/3/PRL/2*PASTOR/4/HEILO/6/PRL/2*PASTOR//PBW343*2/KUKUNA/3/ROLF07 |
| 346 | 7644875 | LTP | IG 41217/4/PRL/2*PASTOR//PBW343*2/KUKUNA/3/ROLF07/5/NELOKI |
| 347 | 7644933 | LTP | IG 122784/4/PRL/2*PASTOR//PBW343*2/KUKUNA/3/ROLF07/5/NELOKI |
| 348 | 7645001 | LTP | H-1698/4/PRL/2*PASTOR//PBW343*2/KUKUNA/3/ROLF07/5/NELOKI |
| 349 | 7645002 | LTP | H-1698/4/PRL/2*PASTOR//PBW343*2/KUKUNA/3/ROLF07/5/NELOKI |
| 350 | 7645006 | LTP | H-1698/4/PRL/2*PASTOR//PBW343*2/KUKUNA/3/ROLF07/5/NELOKI |
| 351 | 7645096 | LTP | ALG86/4/FGO/PALES//MEXI_1/3/RUFF/FGO/5/ENTE/6/AE.SQUARROSA (254)/7/NELOKI/8/ATTILA*2/PBW65//MURGA |
| 352 | 7645132 | LTP | GAN/AE.SQUARROSA (267)//NELOKI/3/ATTILA*2/PBW65//MURGA |
| 353 | 7645139 | LTP | GAN/AE.SQUARROSA (267)//NELOKI/3/ATTILA*2/PBW65//MURGA |
| 354 | 7645140 | LTP | GAN/AE.SQUARROSA (267)//NELOKI/3/ATTILA*2/PBW65//MURGA |
| 355 | 7645154 | LTP | ALG86/4/FGO/PALES//MEXI_1/3/RUFF/FGO/5/ENTE/6/AE.SQUARROSA (723)/7/NELOKI/8/ATTILA*2/PBW65//MURGA |
| 356 | 7645159 | LTP | ALG86/4/FGO/PALES//MEXI_1/3/RUFF/FGO/5/ENTE/6/AE.SQUARROSA (723)/7/NELOKI/8/ATTILA*2/PBW65//MURGA |
| 357 | 7645204 | LTP | INDIA-57/NELOKI/3/ATTILA*2/PBW65//MURGA |
| 358 | 7645227 | LTP | ARVAND 1/3/ATTILA*2/PBW65//MURGA/4/REEDLING #1 |
| 359 | 7645228 | LTP | ARVAND 1/3/ATTILA*2/PBW65//MURGA/4/REEDLING #1 |
| 360 | 7645231 | LTP | ARVAND 1/3/ATTILA*2/PBW65//MURGA/4/REEDLING #1 |
| 361 | 7645251 | LTP | CETA/AE.SQUARROSA (533)/3/ATTILA*2/PBW65//MURGA/4/REEDLING #1 |
| 362 | 7645259 | LTP | GARZA/BOY//AE.SQUARROSA (179)/3/ATTILA*2/PBW65//MURGA/4/REEDLING #1 |
| 363 | 7645275 | LTP | 68.111/RGB-U//WARD RESEL/3/STIL/4/AE.SQUARROSA (617)/5/ATTILA*2/PBW65//MURGA/6/REEDLING #1 |
| 364 | 7645280 | LTP | 68.111/RGB-U//WARD RESEL/3/STIL/4/AE.SQUARROSA (617)/5/ATTILA*2/PBW65//MURGA/6/REEDLING #1 |
| 365 | 7645287 | LTP | 68.111/RGB-U//WARD/3/FGO/4/RABI/5/AE.SQUARROSA (878)/6/ATTILA*2/PBW65//MURGA/7/REEDLING #1 |
| 366 | 7645288 | LTP | 68.111/RGB-U//WARD/3/FGO/4/RABI/5/AE.SQUARROSA (878)/6/ATTILA*2/PBW65//MURGA/7/REEDLING #1 |
| 367 | 7645290 | LTP | 68.111/RGB-U//WARD/3/FGO/4/RABI/5/AE.SQUARROSA (878)/6/ATTILA*2/PBW65//MURGA/7/REEDLING #1 |
| 368 | 7645292 | LTP | 68.111/RGB-U//WARD/3/FGO/4/RABI/5/AE.SQUARROSA (878)/6/ATTILA*2/PBW65//MURGA/7/REEDLING #1 |
| 369 | 7645295 | LTP | 68.111/RGB-U//WARD/3/FGO/4/RABI/5/AE.SQUARROSA (878)/6/ATTILA*2/PBW65//MURGA/7/REEDLING #1 |
| 370 | 7645318 | LTP | IWA8612641/3/ATTILA*2/PBW65//MURGA/4/REEDLING #1 |
| 371 | 7645319 | LTP | IWA8612641/3/ATTILA*2/PBW65//MURGA/4/REEDLING #1 |
| 372 | 7645338 | LTP | IG 41624/REEDLING #1/6/CNO79//PF70354/MUS/3/PASTOR/4/BAV92*2/5/FH6-1-7 |
| 373 | 7645395 | LTP | 68.111/RGB-U//WARD/3/FGO/4/RABI/5/AE.SQUARROSA (809)/6/CNO79//PF70354/MUS/3/PASTOR/4/BAV92*2/5/FH6-1-7/7/KACHU #1/KIRITATI//KACHU |
| 374 | 7645396 | LTP | 68.111/RGB-U//WARD/3/FGO/4/RABI/5/AE.SQUARROSA (809)/6/CNO79//PF70354/MUS/3/PASTOR/4/BAV92*2/5/FH6-1-7/7/KACHU #1/KIRITATI//KACHU |
| 375 | 7645398 | LTP | 68.111/RGB-U//WARD/3/FGO/4/RABI/5/AE.SQUARROSA (809)/6/CNO79//PF70354/MUS/3/PASTOR/4/BAV92*2/5/FH6-1-7/7/KACHU #1/KIRITATI//KACHU |
| 376 | 7645408 | LTP | 68.111/RGB-U//WARD/3/FGO/4/RABI/5/AE.SQUARROSA (809)/6/CNO79//PF70354/MUS/3/PASTOR/4/BAV92*2/5/FH6-1-7/7/KACHU #1/KIRITATI//KACHU |
| 377 | 7645418 | LTP | 68.111/RGB-U//WARD/3/FGO/4/RABI/5/AE.SQUARROSA (809)/6/CNO79//PF70354/MUS/3/PASTOR/4/BAV92*2/5/FH6-1-7/7/KACHU #1/KIRITATI//KACHU |
| 378 | 7645419 | LTP | 68.111/RGB-U//WARD/3/FGO/4/RABI/5/AE.SQUARROSA (809)/6/CNO79//PF70354/MUS/3/PASTOR/4/BAV92*2/5/FH6-1-7/7/KACHU #1/KIRITATI//KACHU |
| 379 | 7645422 | LTP | 68.111/RGB-U//WARD/3/FGO/4/RABI/5/AE.SQUARROSA (809)/6/CNO79//PF70354/MUS/3/PASTOR/4/BAV92*2/5/FH6-1-7/7/KACHU #1/KIRITATI//KACHU |
| 380 | 7645426 | LTP | 68.111/RGB-U//WARD/3/FGO/4/RABI/5/AE.SQUARROSA (809)/6/CNO79//PF70354/MUS/3/PASTOR/4/BAV92*2/5/FH6-1-7/7/KACHU #1/KIRITATI//KACHU |
| 381 | 7645428 | LTP | 68.111/RGB-U//WARD/3/FGO/4/RABI/5/AE.SQUARROSA (809)/6/CNO79//PF70354/MUS/3/PASTOR/4/BAV92*2/5/FH6-1-7/7/KACHU #1/KIRITATI//KACHU |
| 382 | 7645476 | LTP | DOY1/AE.SQUARROSA (318)/3/KACHU #1/KIRITATI//KACHU/4/PBW343*2/KUKUNA*2//FRTL/PIFED |
| 383 | 7645493 | LTP | QRO94.1.4/3/KACHU #1/KIRITATI//KACHU/4/PBW343*2/KUKUNA*2//FRTL/PIFED |
| 384 | 7645495 | LTP | QRO94.1.4/3/KACHU #1/KIRITATI//KACHU/4/PBW343*2/KUKUNA*2//FRTL/PIFED |
| 385 | 7645500 | LTP | QRO94.1.4/3/KACHU #1/KIRITATI//KACHU/4/PBW343*2/KUKUNA*2//FRTL/PIFED |
| 386 | 7645514 | LTP | JAL95.4.3/3/KACHU #1/KIRITATI//KACHU/4/PBW343*2/KUKUNA*2//FRTL/PIFED |
| 387 | 7645614 | LTP | D67.2/PARANA 66.270//AE.SQUARROSA (354)/3/KACHU #1/KIRITATI//KACHU/4/PBW343*2/KUKUNA*2//FRTL/PIFED |
| 388 | 7645615 | LTP | D67.2/PARANA 66.270//AE.SQUARROSA (354)/3/KACHU #1/KIRITATI//KACHU/4/PBW343*2/KUKUNA*2//FRTL/PIFED |
| 389 | 7645622 | LTP | D67.2/PARANA 66.270//AE.SQUARROSA (354)/3/KACHU #1/KIRITATI//KACHU/4/PBW343*2/KUKUNA*2//FRTL/PIFED |
| 390 | 7645662 | LTP | YAV_2/TEZ//AE.SQUARROSA (249)/3/KACHU #1/KIRITATI//KACHU/4/PBW343*2/KUKUNA*2//FRTL/PIFED |
| 391 | 7645680 | LTP | IRAQ-50/3/KACHU #1/KIRITATI//KACHU/4/PBW343*2/KUKUNA*2//FRTL/PIFED |
| 392 | 7645690 | LTP | IG 41237/3/PBW343*2/KUKUNA*2//FRTL/PIFED/4/QUAIU #1 |
| 393 | 7645702 | LTP | IG 41237/3/PBW343*2/KUKUNA*2//FRTL/PIFED/4/QUAIU #1 |
| 394 | 7645781 | LTP | W98/3/PBW343*2/KUKUNA*2//FRTL/PIFED/4/QUAIU #1 |
| 395 | 7645787 | LTP | W98/3/PBW343*2/KUKUNA*2//FRTL/PIFED/4/QUAIU #1 |
| 396 | 7645798 | LTP | W98/3/PBW343*2/KUKUNA*2//FRTL/PIFED/4/QUAIU #1 |
| 397 | 7645809 | LTP | W98/3/PBW343*2/KUKUNA*2//FRTL/PIFED/4/QUAIU #1 |
| 398 | 7645860 | LTP | OAX93.10.1/ROLF07//NAVJ07 |
| 399 | 7645878 | LTP | AE.SQUARROSA (1031)/DVERD_2//NAVJ07/3/KACHU |
| 400 | 7645883 | LTP | AE.SQUARROSA (1031)/DVERD_2//NAVJ07/3/KACHU |
| 401 | 7645891 | LTP | 68.111/RGB-U//WARD RESEL/3/STIL/4/AE.SQUARROSA (675)/5/NAVJ07/6/KACHU |
| 402 | 7645903 | LTP | 68.111/RGB-U//WARD RESEL/3/STIL/4/AE.SQUARROSA (675)/5/NAVJ07/6/KACHU |
| 403 | 7645906 | LTP | 68.111/RGB-U//WARD/3/FGO/4/RABI/5/AE.SQUARROSA (675)/6/NAVJ07/7/KACHU |
| 404 | 7645912 | LTP | 68.111/RGB-U//WARD/3/FGO/4/RABI/5/AE.SQUARROSA (675)/6/NAVJ07/7/KACHU |
| 405 | 7645914 | LTP | 68.111/RGB-U//WARD/3/FGO/4/RABI/5/AE.SQUARROSA (675)/6/NAVJ07/7/KACHU |
| 406 | 7645915 | LTP | 68.111/RGB-U//WARD/3/FGO/4/RABI/5/AE.SQUARROSA (675)/6/NAVJ07/7/KACHU |
| 407 | 7645960 | LTP | IG 41468/NAVJ07//KACHU |
| 408 | 7645961 | LTP | 68.111/RGB-U//WARD/3/AE.SQUARROSA (452)/4/2*OASIS/SKAUZ//4*BCN/5/NAVJ07/6/KACHU |
| 409 | 7645964 | LTP | 68.111/RGB-U//WARD/3/AE.SQUARROSA (452)/4/2*OASIS/SKAUZ//4*BCN/5/NAVJ07/6/KACHU |
| 410 | 7645978 | LTP | 68.111/RGB-U//WARD/3/AE.SQUARROSA (452)/4/2*OASIS/SKAUZ//4*BCN/5/NAVJ07/6/KACHU |
| 411 | 7645979 | LTP | 68.111/RGB-U//WARD/3/AE.SQUARROSA (452)/4/2*OASIS/SKAUZ//4*BCN/5/NAVJ07/6/KACHU |
| 412 | 7645982 | LTP | 68.111/RGB-U//WARD/3/AE.SQUARROSA (452)/4/2*OASIS/SKAUZ//4*BCN/5/NAVJ07/6/KACHU |
| 413 | 7645987 | LTP | 68.111/RGB-U//WARD/3/AE.SQUARROSA (452)/4/2*OASIS/SKAUZ//4*BCN/5/NAVJ07/6/KACHU |
| 414 | 7645997 | LTP | 68.111/RGB-U//WARD/3/AE.SQUARROSA (452)/4/2*OASIS/SKAUZ//4*BCN/5/NAVJ07/6/KACHU |
| 415 | 7646006 | LTP | 68.111/RGB-U//WARD/3/AE.SQUARROSA (452)/4/2*OASIS/SKAUZ//4*BCN/5/NAVJ07/6/KACHU |
| 416 | 7646007 | LTP | 68.111/RGB-U//WARD/3/AE.SQUARROSA (452)/4/2*OASIS/SKAUZ//4*BCN/5/NAVJ07/6/KACHU |
| 417 | 7646008 | LTP | 68.111/RGB-U//WARD/3/AE.SQUARROSA (452)/4/2*OASIS/SKAUZ//4*BCN/5/NAVJ07/6/KACHU |
| 418 | 7646011 | LTP | 68.111/RGB-U//WARD/3/AE.SQUARROSA (452)/4/2*OASIS/SKAUZ//4*BCN/5/NAVJ07/6/KACHU |
| 419 | 7646031 | LTP | 68.111/RGB-U//WARD/3/AE.SQUARROSA (452)/4/2*OASIS/SKAUZ//4*BCN/5/NAVJ07/6/KACHU |
| GID = line identifier; LN = group of the population | | | |

**Table Sup. 2**. Races of yellow rust in cycles FW 2015-16 and SS 2016

| Pst race^a^ | Avirulence/virulence |
| --- | --- |
| MEX96.11 | *YR,1,8,17,POLL/YR2,3,6,7,9,27,A* |
| MEX10.44 | *YR3,8,27,POLL/YR1,2,6,7,9,17,31,A* |
| MEX14.191 | *YR1.POLL/YR2,6,7,8,9,17,27,31,32,A* |
| MEX14.146 | *POLL/YR1,2,3,6,7,8,9,17,31,32,A* |
| MEX14.141 | *Yr5,10,15,24,26,Sp,Poll/YR1,2,3,6,7,8,9,17,27,31,32,A* |

| **Table Sup. 3.** Summary, heritability statistics and BLUPs of YR in five locations | | | | | | |
| --- | --- | --- | --- | --- | --- | --- |
| **Statistic** | **Locations** | **Celaya** | **Jalisco** | **Texcoco** | **Tlaxcala** | **Villagrán** |
| Range % | 2.5-56 | 0-70 | 0-35 | 2.5-70 | 5-100 | 0-50 |
| Mean | 17.554 | 16.244 | 6.704 | 20.673 | 34.234 | 9.915 |
| Heritability | 0.842 | 0.943 | 0.981 | 0.932 | 0.944 | 0.966 |
| Genotype Variance | 95.07 | 181.462 | 40.257 | 156.465 | 401.848 | 89.314 |
| GenxLoc Variance | 79.208 |  |  |  |  |  |
| Residual Variance | 19.776 | 22.101 | 1.548 | 22.729 | 47.253 | 6.303 |
| LSD | 7.653 | 6.532 | 1.798 | 6.568 | 9.415 | 3.664 |
| CV | 25.333 | 28.941 | 18.557 | 23.061 | 20.08 | 25.321 |
| n Replicates | 2 | 2 | 2 | 2 | 2 | 2 |
| n Environments | 5 |  |  |  |  |  |
| Genotype significance | 5.09E-162 | 1.69E-137 | 1.45E-227 | 2.23E-123 | 1.68E-137 | 1.11E-169 |
| GenxEnv significance | 0 |  |  |  |  |  |
|  |  |  |  |  |  |  |

**Table Sup. 4.** Sequence and chromosomal location of the significant SNPs

| **SNPs** | **Sequence** | **Alleles** | **Chr** |
| --- | --- | --- | --- |
| 100272191\|F\|0-37:C>G-37:C>G | TGCAGTTGTGTAAGGGTGCCCTCTGTGAGGTTCACCT**C**GAACACTGCATTGCACACGGCTAGGTTGCCG | C/G | 2A |
| 1177572\|F\|0-15:T>G-15:T>G | TGCAGTTTACGAGAT**T**CAGGTGGCTCAGGCTCGCCAGCAGGTTGCTCTCCAACCAGCTAGGCGACGATG | T/G | 2A |
| 1206128\|F\|0-54:G>A-54:G>A | TGCAGGTACGTATGTGAATCAAATCAATCACCAGTGCTCTTTTTGTTCCACTCG**G**TACTATGCTTTCCC | G/A | 2A |
| 1028859\|F\|0-50:G>C-50:G>C | TGCAGATGCCGAGCTCACGGAGATGGGGGAAGGAGGTGCCGAGCGGTAAC**G**TGGCCACGTCCGAGATCG | G/C | 2A |
| 1088511\|F\|0-7:T>C-7:T>C | TGCAGTG**T**TTCCTCCACTCTCTCCACAGGGTCCCTATCTTCCCCCAGGATCACAACCCCTTTCTCCGAG | T/C | 2A |
| 1092886\|F\|0-39:A>C-39:A>C | TGCAGCCTGGACGCCAGGGGCGGCCTGCTCGCGCGCTAC**A**TCAGGGCGTCGTCCGCCCACCTCGGCAAG | A/C | 2A |
| 7940374\|F\|0-14:A>C-14:A>C | TGCAGGGTGTGGTC**A**GAGCGAGCCACCACGAAGAGCGCGCCCTCCCCGTTGCAGTTGACCTCGGTTCGT | A/C | 2A |
| 3951942\|F\|0-8:A>G-8:A>G | TGCAGCTC**A**TGTCCAACGACAGGTTCAGGAGCGTCGAGCACCGCGTGGTGTCCAACGGCGTGGGGCCAC | A/G | 2A |
| 100247987\|F\|0-40:G>A-40:G>A | TGCAGTTCTTGAGGTACAGGCGCGTCAGCTTCTCCAGTCC**G**CTGAGCCAGGTAGGCGAGGTCTCCCCGA | G/A | 2A |
| 2293684\|F\|0-18:C>T-18:C>T | TGCAGGCAGCGGATTACG**C**GCGTGGTGCGTCGCTTTGTCAAACCGATCGAGCGACAAAAAAGTCACTCG | C/T | 2A |
| 5411524\|F\|0-14:G>A-14:G>A | TGCAGGAACTGGCG**G**GTGCTGCCATGCCTGAGCCAGCTTCCTGACCTCAGAGTCCTGCACATCAAAGAG | G/A | 2B |
| 1030280\|F\|0-14:G>C-14:G>C | TGCAGGATGTTGTC**G**GTGCTCCACAGGCTGCCGTCGTCGGCGTCGTCGCAGCCGCCGCAGTGGCTGAAA | G/C | 2D |
| 1004337\|F\|0-6:C>T-6:C>T | TGCAGT**C**GGGTAAGTGTGCCCTCTGTGAGGTTCACCTGGAACACTGCATGGCACACGGCTAGGCAGCCG | C/T | 2D |
| 5324283\|F\|0-29:A>G-29:A>G | TGCAGCATGACGAGGATGAGGCAGACCAC**A**ACCGACGACGCGAGCGCGGTGCCGTTGCAGTAGTAGAAG | A/G | 2D |

In the marker sequence, the red nitrogenous base indicates the change of the SNP.

**Table Sup. 5.** Distribution of SNPs in resistant lines.

|  |  | M1 | M2 | M3 | M4 | M5 | M6 | M7 | M8 | M9 | M10 | M11 | M12 | M13 | M14 |
| --- | --- | --- | --- | --- | --- | --- | --- | --- | --- | --- | --- | --- | --- | --- | --- |
| YR (%) | GID | T>**G** | **C**>G | **G**>A | **G**>C | T>**C** | A>**C** | **A**>C | A>**G** | G>**A** | C>**T** | **G**>A | **G**>C | **C**>T | **A**>G |
| 4 | 7645288 | X | NN | X | X | X | X | X | X | X | X | X | X | X | X |
| 4 | 7643155 | X | X | X | X | X | X | X | X | X | X | X | X | X | X |
| 4 | 7643287 | X | X | X | X | X | X | X | X | X | X | X | X | X | X |
| 4.5 | 7645290 | X | x | X | X | X | X | X | X | X | X | X | X | x | X |
| 4.5 | 7643383 | NN | X | X | X | X | X | X | X | X | X | X | X | X | X |
| 5 | 7646008 | X | X | X | X | X | X | X | X | X | X | X | X | X | X |
| 5 | 7641373 | X | X | X | X | X | X | X | X | X | X | X | X | X | X |
| 5 | 7643156 | X | X | X | X | X | X | X | NN | X | X | X | X | X | X |
| 5 | 7643139 | X | X | X | X | X | X | X | X | X | X | X | X | X | X |
| 5 | 7643152 | X | X | X | X | X | X | X | X | X | X | X | X | X | X |
| 5 | 7643478 | X | X | X | X | X | X | X | X | X | X | X | X | X | X |
| 5.1 | 7644145 | X | X | X | X | X | X | X | X | X | X | X | X | X | X |
| 5.1 | 7645476 | X | X | X | x | X | X | X | X | X | X | X | x | X | X |
| 5.5 | 7642809 | X | X | X | X | X | X | X | X | X | X | X | X | X | X |
| 5.5 | 7642941 | NN | X | NN | X | X | X | X | X | X | X | X | X | X | NN |
| 5.5 | 7643333 | X | X | X | X | X | X | X | X | X | X | X | X | X | X |
| 5.5 | 7643276 | X | X | X | X | X | X | X | X | X | X | X | X | X | X |
| 5.5 | 7643253 | X | X | X | X | X | X | X | X | X | X | X | X | X | X |
| 5.5 | 7643272 | X | X | X | X | X | X | X | X | X | X | X | X | X | X |
| 5.5 | 7642734 | X | X | X | X | X | X | X | X | X | X | X | X | X | X |

YR = Percentage of yellow rust; GID = line identifier; M= marker, X=presence of resistant allele; - absence of resistant allele; N=lost data; M1 = 1177572|F|0-15:T>G-15:T>G; M2 = 100272191|F|0-37:C>G-37:C>G; M3 = 1206128|F|0-54:G>A-54:G>A; M4 = 1028859|F|0-50:G>C-50:G>C; M5 = 1088511|F|0-7:T>C-7:T>C; M6 = 1092886|F|0-39:A>C-39:A>C; M7 = 7940374|F|0-14:A>C-14:A>C; M8 = 3951942|F|0-8:A>G-8:A>G; M9 = 100247987|F|0-40:G>A-40:G>A; M10 = 2293684|F|0-18:C>T-18:C>T; M11 = 5411524|F|0-14:G>A-14:G>A; M12 = 1030280|F|0-14:G>C-14:G>C; M13 = 1004337|F|0-6:C>T-6:C>T; M14 = 5324283|F|0-29:A>G-29:A>G.

**Table Sup. 6.** Markers in susceptible lines

|  |  | M1 | M2 | M3 | M4 | M5 | M6 | M7 | M8 | M9 | M10 | M11 | M12 | M13 | M14 |
| --- | --- | --- | --- | --- | --- | --- | --- | --- | --- | --- | --- | --- | --- | --- | --- |
| YR (%) | GID | T>**G** | **C**>G | **G**>A | **G**>C | T>**C** | A>**C** | **A**>C | A>**G** | G>**A** | C>**T** | **G**>A | **G**>C | **C**>T | **A**>G |
| 36 | 7642498 | - | - | - | NN | - | - | - | - | - | - | - | - | - | - |
| 36 | 7641911 | - | - | - | - | - | - | - | - | - | - | - | - | - | - |
| 37 | 7641971 | - | - | - | - | - | - | - | - | - | - | - | - | - | - |
| 38.5 | 7640864 | - | - | - | - | - | - | - | - | - | - | - | - | - | - |
| 39 | 7642587 | - | - | - | - | - | - | - | - | - | - | - | - | - | - |
| 40 | 7642500 | - | - | - | NN | - | - | - | - | - | - | - | - | - | - |
| 41 | 7641951 | - | - | - | - | - | - | - | NN | - | - | - | - | - | - |
| 41.5 | 7640926 | - | - | - | - | - | - | - | - | - | - | - | - | - | NN |
| 42 | 7642204 | - | - | - | NN | - | - | - | - | - | - | - | - | - | - |
| 43 | 7640794 | - | - | - | - | x | - | - | - | - | - | - | - | - | - |
| 43 | 7644526 | - | - | - | NN | - | - | - | - | - | - | - | - | - | - |
| 43 | 7644415 | - | - | - | NN | - | - | - | - | - | - | - | - | - | NN |
| 43 | 7641917 | - | - | - | - | - | - | - | - | - | NN | - | - | - | - |
| 44 | 7641906 | - | - | - | - | - | - | - | - | - | - | - | - | - | - |
| 44.5 | 7640852 | - | - | - | - | - | - | - | - | - | - | - | - | - | - |
| 45 | 7642238 | - | - | - | - | - | - | - | - | - | NN | - | - | - | - |
| 47.5 | 7642707 | - | - | - | NN | - | - | NN | - | - | - | - | - | - | - |
| 48 | 7642281 | - | - | - | - | - | - | - | - | - | - | - | - | - | - |
| 51 | 7641909 | - | - | - | - | - | - | - | - | - | - | - | - | - | - |
| 54 | 7642418 | - | - | - | - | - | - | - | - | - | NN | - | - | - | NN |

% of Yr= Percentage of yellow rust; LN= group of the population; GID = line identifier; M= marker, X=presence of resistant allele; - absence of resistant allele; N=lost data; M1 = 1177572|F|0-15:T>G-15:T>G; M2 = 100272191|F|0-37:C>G-37:C>G; M3 = 1206128|F|0-54:G>A-54:G>A; M4 = 1028859|F|0-50:G>C-50:G>C; M5 = 1088511|F|0-7:T>C-7:T>C; M6 = 1092886|F|0-39:A>C-39:A>C; M7 = 7940374|F|0-14:A>C-14:A>C; M8 = 3951942|F|0-8:A>G-8:A>G; M9 = 100247987|F|0-40:G>A-40:G>A; M10 = 2293684|F|0-18:C>T-18:C>T; M11 = 5411524|F|0-14:G>A-14:G>A; M12 = 1030280|F|0-14:G>C-14:G>C; M13 = 1004337|F|0-6:C>T-6:C>T; M14 = 5324283|F|0-29:A>G-29:A>G.

**Table Sup. 7. Blast results of SNPs associated with yellow rust resistance in PBLs**

| Marker | Chr | Position in Wheat genome (IWGSC) | Candidate gene hit:direct | Candidate gene hit:closest | Description | Gene structure | Molecular Function |
| --- | --- | --- | --- | --- | --- | --- | --- |
| 100272191\|F\|0-37:C>G-37:C>G | 2A | 18,495,146-18,495,221 | [TraesCS2A02G047700.1](https://plants.ensembl.org/Triticum_aestivum/Transcript/Summary?db=core;g=TraesCS2A02G047700;r=2A:18494069-18495282;t=TraesCS2A02G047700.1;tl=sui1vAqcQBjhk1AT-19337062-863200414) |  | This gene has 1 transcript (splice variant), 5 orthologues and 1 paralogue. | Exons: 2, Coding exons: 2, Transcript length: 1,053 bps, Translation length: 350 residues |  |
| 1177572\|F\|0-15:T>G-15:T>G | 2A | 13,437,985-13,438,060 | [TraesCS2A02G029900](https://plants.ensembl.org/Triticum_aestivum/Gene/Summary?db=core;g=TraesCS2A02G029900;r=2A:18494069-18495282;tl=Z9akApyzzuN86QyX-19337067-863200948) |  | This gene has 1 transcript (splice variant), 10 orthologues and 737 paralogues. | Exons: 1, Coding exons: 1, Transcript length: 3,675 bps, Translation length: 1,080 residues |  |
| 1206128\|F\|0-54:G>A-54:G>A | 2A | 14,215,341-14,215,416 | [TraesCS2A02G030800](https://plants.ensembl.org/Triticum_aestivum/Gene/Summary?db=core;g=TraesCS2A02G030800;r=2A:18494069-18495282;tl=klMpgRIpF9uT1BJ4-19337072-863201202) |  | This transcript has 2 exons, is annotated with 5 domains and features and is associated with 88 variant alleles. | Exons: 2, Coding exons: 2, Transcript length: 849 bps, Translation length: 244 residues |  |
| 1028859\|F\|0-50:G>C-50:G>C | 2A | 8,143,117-8,143,186 |  | [TraesCS2A02G017300.1](https://plants.ensembl.org/Triticum_aestivum/Transcript/Summary?db=core;g=TraesCS2A02G017300;r=2A:8141118-8143112;t=TraesCS2A02G017300.1) | This gene has 1 transcript (splice variant), 10 orthologues and 10 paralogues. | Exons: 3, Coding exons: 3, Transcript length: 867 bps, Translation length: 288 residues | FBD domain |
| 1088511\|F\|0-7:T>C-7:T>C | 2A | 16,730,047-16,730,120 | [TraesCS2A02G041400.1](https://plants.ensembl.org/Triticum_aestivum/Transcript/Summary?db=core;g=TraesCS2A02G041400;r=2A:16729086-16741609;t=TraesCS2A02G041400.1;tl=Ba3d2XdHR3PjfMio-19339461-863782788) |  | This gene has 1 transcript (splice variant), 87 orthologues and 12 paralogues. | Exons: 19, Coding exons: 19, Transcript length: 2,180 bps, Translation length: 608 residues | Amidase |
| 1092886\|F\|0-39:A>C-39:A>C | 2A | 16,016,618-16,016,693 | [TraesCS2A02G038100.1](https://plants.ensembl.org/Triticum_aestivum/Transcript/Summary?db=core;g=TraesCS2A02G038100;r=2A:16729086-16741609;t=TraesCS2A02G038100.1;tl=R4axuEwmvYriihVW-19339467-863782911) |  | This gene has 1 transcript (splice variant), 107 orthologues and 30 paralogues. | Exons: 1, Coding exons: 1, Transcript length:1,915 bps, Translation length: 590 residues | D-arabinono-1,4-lactone oxidase |
| 7940374\|F\|0-14:A>C-14:A>C | 2A | 16,630,180-16,630,248 | [TraesCS2A02G040800.1](https://plants.ensembl.org/Triticum_aestivum/Transcript/Summary?db=core;g=TraesCS2A02G040800;r=2A:16729086-16741609;t=TraesCS2A02G040800.1;tl=c6v03JBhZoi5W6Qn-19339470-863782961) |  | This gene has 1 transcript (splice variant), 278 orthologues and 88 paralogues. | Exons: 2, Coding exons: 2, Transcript length:1,615 bps, Translation length: 452 residues | Chloramphenicol acetyltransferase-like domain superfamily |
| 3951942\|F\|0-8:A>G-8:A>G | 2A | 15,960,617-15,960,692 | [TraesCS2A02G037900.1](https://plants.ensembl.org/Triticum_aestivum/Transcript/Summary?db=core;g=TraesCS2A02G037900;r=2A:16729086-16741609;t=TraesCS2A02G037900.1;tl=2XWk6zTubY5I5oEl-19339472-863783023) |  | This transcript has 2 exons, is annotated with 10 domains and features, is associated with 13 variant alleles and maps to 1 oligo probe. | Exons: 2, Coding exons: 2, Transcript length:1,068 bps, Translation length: 355 residues | Isopenicillin N synthase-like, Oxoglutarate/iron-dependent dioxygenase |
| 100247987\|F\|0-40:G>A-40:G>A | 2A | 13,252,201-13,252,275 | [TraesCS2A02G029700.1](https://plants.ensembl.org/Triticum_aestivum/Transcript/Summary?db=core;g=TraesCS2A02G029700;r=2A:16729086-16741609;t=TraesCS2A02G029700.1;tl=NN2U47Zjes2Ioa4F-19339476-863783420) |  | This gene has 1 transcript (splice variant), 5 orthologues and 737 paralogues. | Exons: 2, Coding exons: 1, Transcript length:3,215 bps, Translation length: 922 residues | NB-ARC, P-loop containing nucleoside triphosphate hydrolase |
| 2293684\|F\|0-18:C>T-18:C>T | 2A | 14,418,706-14,418,781 |  | TraesCS2A02G031200.1 | This gene has 1 transcript (splice variant), 5 orthologues and 5 paralogues. | Exons: 3, Coding exons: 3, Transcript length: 2,141 bps, Translation length: 592 residues | Cullin repeat-like-containing domain superfamily, Exocyst complex component Exo70 |
| 5411524\|F\|0-14:G>A-14:G>A | 2B | 22,307,593-22,307,667 | [TraesCS2B02G045300.1](https://plants.ensembl.org/Triticum_aestivum/Transcript/Summary?db=core;g=TraesCS2B02G045300;r=2A:14418706-14418781;t=TraesCS2B02G045300.1;tl=Qx36EKJ7QKbP5e8w-19339481-863802020) |  | This transcript has 1 exon, is annotated with 20 domains and features and is associated with 570 variant alleles. | Exons: 1, Coding exons: 1, Transcript length: 3,608 bps, Translation length: 1,064 residues | Leucine-rich repeat domain superfamily |
| 1030280\|F\|0-14:G>C-14:G>C | 2D | 273,830-273,905 | [TraesCS2D02G000500.1](https://plants.ensembl.org/Triticum_aestivum/Transcript/Summary?db=core;g=TraesCS2D02G000500;r=2A:14418706-14418781;t=TraesCS2D02G000500.1;tl=bOop25PuMwiTNnmf-19339483-863802087) |  | This transcript has 2 exons, is annotated with 25 domains and features, is associated with 324 variant alleles and maps to 27 oligo probes. | Exons: 2, Coding exons: 2, Transcript length: 2,810 bps, Translation length: 776 residues | Ethylene receptor, GAF domain |
| 1004337\|F\|0-6:C>T-6:C>T | 2D | 16,959,380-16,959,455 | [TraesCS2D02G046300.1](https://plants.ensembl.org/Triticum_aestivum/Transcript/Summary?db=core;g=TraesCS2D02G046300;r=2A:14418706-14418781;t=TraesCS2D02G046300.1;tl=xznXMgyz0XI9159A-19339485-863802225) |  | This gene has 1 transcript (splice variant), 5 orthologues and 5 paralogues. | Exons: 2, Coding exons: 2, Transcript length: 1,062 bps, Translation length: 353 residues |  |
| 5324283\|F\|0-29:A>G-29:A>G | 2D | 14,744,316-14,744,391 |  | [TraesCS2D02G040300.1](https://plants.ensembl.org/Triticum_aestivum/Transcript/Summary?db=core;g=TraesCS2D02G040300;r=2D:14711769-14712380;t=TraesCS2D02G040300.1) | This gene has 1 transcript (splice variant), 89 orthologues and 43 paralogues. | Exons: 1, Coding exons: 1, Transcript length: 612 bps, Translation length: 203 residues | AP2/ERF domain |


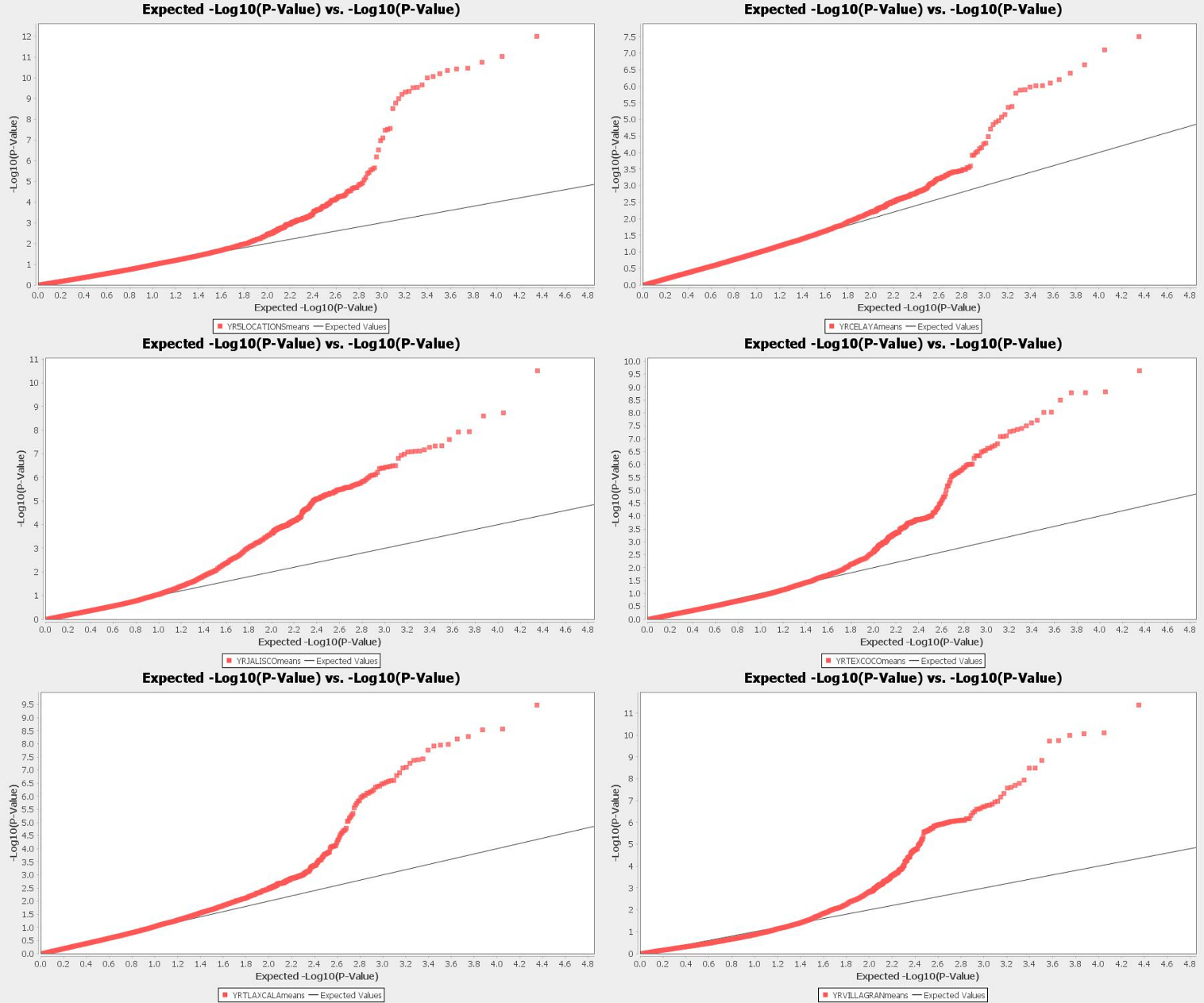


**Fig. Sup. 1.** Q-Q Plot of the association of 419 lines with the yellow rust in five locations and across 5 locations combined.


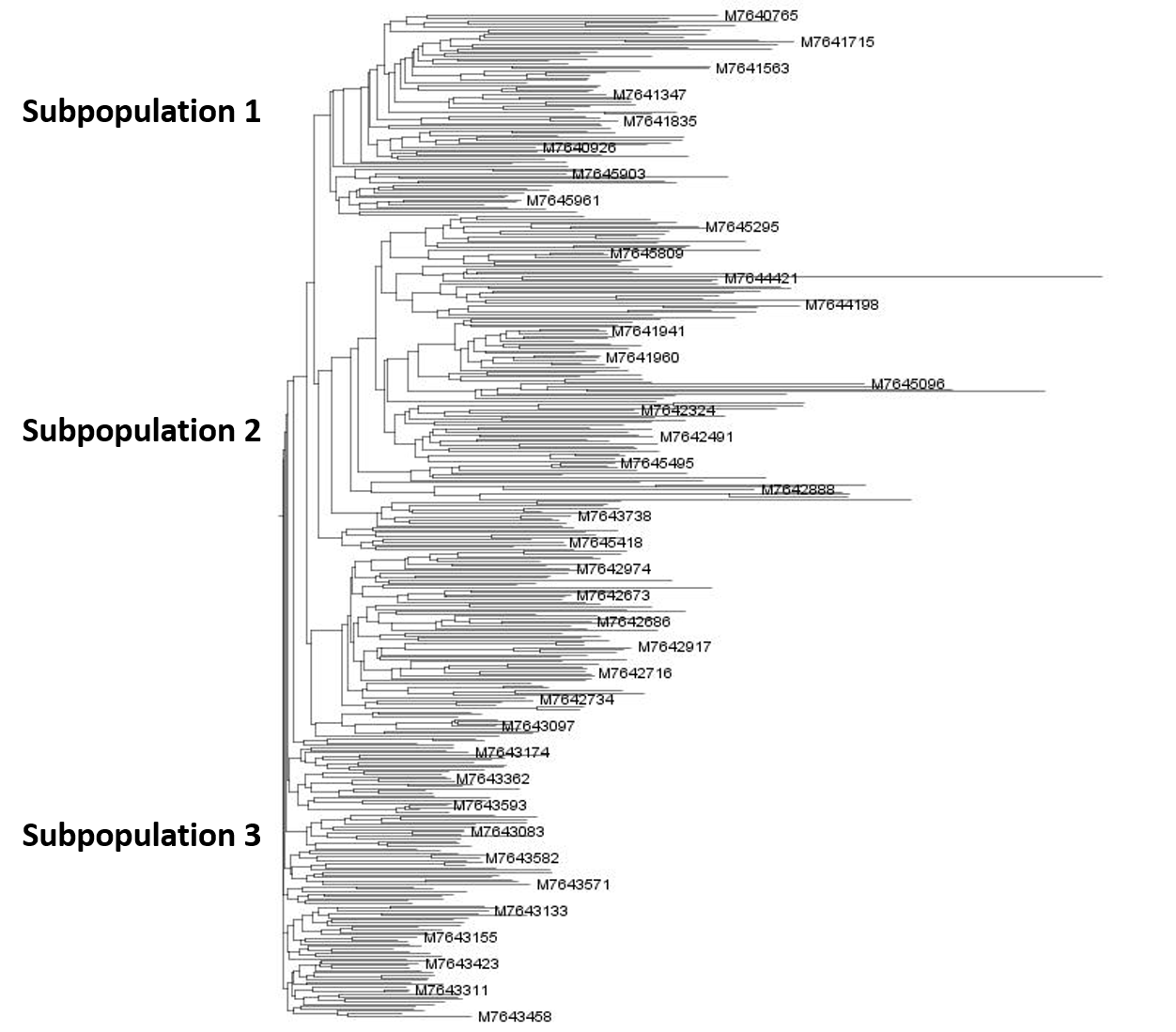


**Fig. Sup. 2.** Cladogram of the 419 lines with the 22415 markers
